# TcpC inhibits neutrophil extracellular trap formation by enhancing ubiquitination mediated degradation of peptidylarginine deiminase 4

**DOI:** 10.1038/s41467-021-23881-8

**Published:** 2021-06-09

**Authors:** Qian Ou, Jia-qi Fang, Zhe-sheng Zhang, Zhe Chi, Jie Fang, Di-yan Xu, Kai-zhong Lu, Meng-qing Qian, Da-yong Zhang, Jun-ping Guo, Wei Gao, Na-ru Zhang, Jian-ping Pan

**Affiliations:** 1 Institute of Translational Medicine, Zhejiang University City College, Hangzhou, P. R. China; 2 Department of Basic Medical Sciences, Zhejiang University School of Medicine, Hangzhou, P. R. China; 3 Department of Clinical Medicine, Zhejiang University City College School of Medicine, Hangzhou, P. R. China

**Keywords:** Infection, Neutrophils, Bacterial immune evasion, Experimental models of disease

## Abstract

TcpC is a multifunctional virulence factor of uropathogenic *E. coli* (UPEC). Neutrophil extracellular trap formation (NETosis) is a crucial anti-infection mechanism of neutrophils. Here we show the influence of TcpC on NETosis and related mechanisms. We show NETosis in the context of a pyelonephritis mouse model induced by TcpC-secreting wild-type *E. coli* CFT073 (CFT073^wt^) and LPS-induced in vitro NETosis with CFT073^wt^ or recombinant TcpC (rTcpC)-treated neutrophils are inhibited. rTcpC enters neutrophils through caveolin-mediated endocytosis and inhibits LPS-induced production of ROS, proinflammatory cytokines and protein but not mRNA levels of peptidylarginine deiminase 4 (PAD4). rTcpC treatment enhances PAD4 ubiquitination and accumulation in proteasomes. Moreover, in vitro ubiquitination kit analyses show that TcpC is a PAD4-targetd E3 ubiquitin-ligase. These data suggest that TcpC inhibits NETosis primarily by serving as an E3 ligase that promotes degradation of PAD4. Our findings provide a novel mechanism underlying TcpC-mediated innate immune evasion.

## Abstract (web summary)

TcpC is a well characterised multifunctional virulence factor expressed by uropathogenic *Eschericia coli*. Here the authors show that TcpC also targets neutrophil NETosis via its E3 ligase functionality promoting the degradation of PAD4, and represents an additional immune evasion function of this bacterially derived virulence factor.

## Introduction

Urinary tract infection (UTI) is one of the most common bacterial infections affecting 150 million people worldwide each year^1,2^. It is estimated that the medical burden of UTI exceeds $6 billion annually^3^. These significant infections can be a serious health problem to reduce the quality of life. *E. coli* is the most common pathogen of UTIs, and ~80% of UTI are caused by *E. coli*^3^. Urinary pathogenic *E. coli* (UPEC) can specifically adhere to and implant into urothelial mucosal epithelial cells, leading to UTIs, including asymptomatic bacteriuria, urethritis, urocystitis, and pyelonephritis (PN)^4^.

It was found that most strains of UPEC secrete a protein containing the Toll/interleukin 1 receptor (TIR) domain or TcpC^5^. TcpC is an important virulence factor of UPEC that subverts the toll like receptor (TLR) signaling pathway by direct association of myeloid differentiation factor 88 (MyD88)^5,6^. TcpC inhibits the bactericidal activity of macrophages and promotes the survival of UPEC in the host, which favors the pathogenicity of the pathogen^5,7^.

The characteristic pathological changes of acute pyelonephritis are neutrophil infiltration^5^. It is well-known that neutrophils are important effector arms of the innate immune system. Neutrophils play crucial roles in clearance of extracellular bacterial infection by phagocytosis. Over the past years, increasing evidence has demonstrated that neutrophils have, in addition to phagocytosis, a special anti-infection mechanism that produces neutrophil extracellular traps (NETs), the process of NETs formation is referred to as NETosis^8^. NETs are three dimensional meshwork consisting of chromatin, antimicrobial components such as myeloperoxidase (MPO) and neutrophil elastase (NE)^9^. These fibrous networks can catch and eliminate bacteria, fungi, and viral particles as well^9–11^. NETosis can be induced by a wide range of substances, including bacteria, lipopolyssacharide (LPS), phorbol-12-myristate-13-acetate (PMA), and viruses, etc^12^. The specific requirements for NETs formation depend on the stimulus, but histone citrullination mediated by the peptidylarginine deiminase 4 (PAD4) has been demonstrated to be an essential step in NETosis^9,13–15^.

PAD4 is primarily expressed in neutrophils, it catalyzes the conversion of histone arginine to citrullination^16^. Citrullination is a post-translational modification that plays an important role in many physiological processes such as cell differentiation and renewing, but abnormal citrullination, however, can lead to the development of diseases, including autoimmune diseases, cancers, and cardiovascular diseases^13^. Numerous stimuli have been confirmed to activate PAD4 and NETosis^17,18^.

There are reactive oxygen species (ROS)-dependent and ROS-independent NETosis. ROS-dependent NETs release requires the production of ROS by NADPH oxidase^19^. ROS stimulate MPO to trigger the activation and translocation of NE from the azurophilic granules to the nucleus where NEs digest nucleosomal histones and promote chromatin relaxation, which favors the citrullination of histone (CitH3) by PAD4^20^. Citrullination of histone leads to chromatin decondensation, which causes entropic chromatin swelling^9^. Subsequently, MPO associate with chromatin and, synergistically with NE and PAD4, promote massive chromatin decondensation^15^. In association with diverse granular and cytoplasmic proteins, decondensed chromatin is eventually released into the extracellular space^17^.

Although TcpC was demonstrated to play a crucial role in the immune evasion of macrophage-mediated innate immunity^5,6,21^, its influence on NETosis remains elusive. In the present study, we show that TcpC suppresses NETosis by promoting degradation of PAD4 through ubiquitin proteasome pathway. Our findings provide not only a novel mechanism underlying TcpC-mediated innate immune evasion, but also new insight into the pathogenicity of pathological microbes.

## Results

### NETosis is inhibited in PN mouse model induced by TcpC-secreting wild-type CFT073 (CFT073^wt^) and in CFT073^wt^-treated neutrophils

**Fig. 1:**
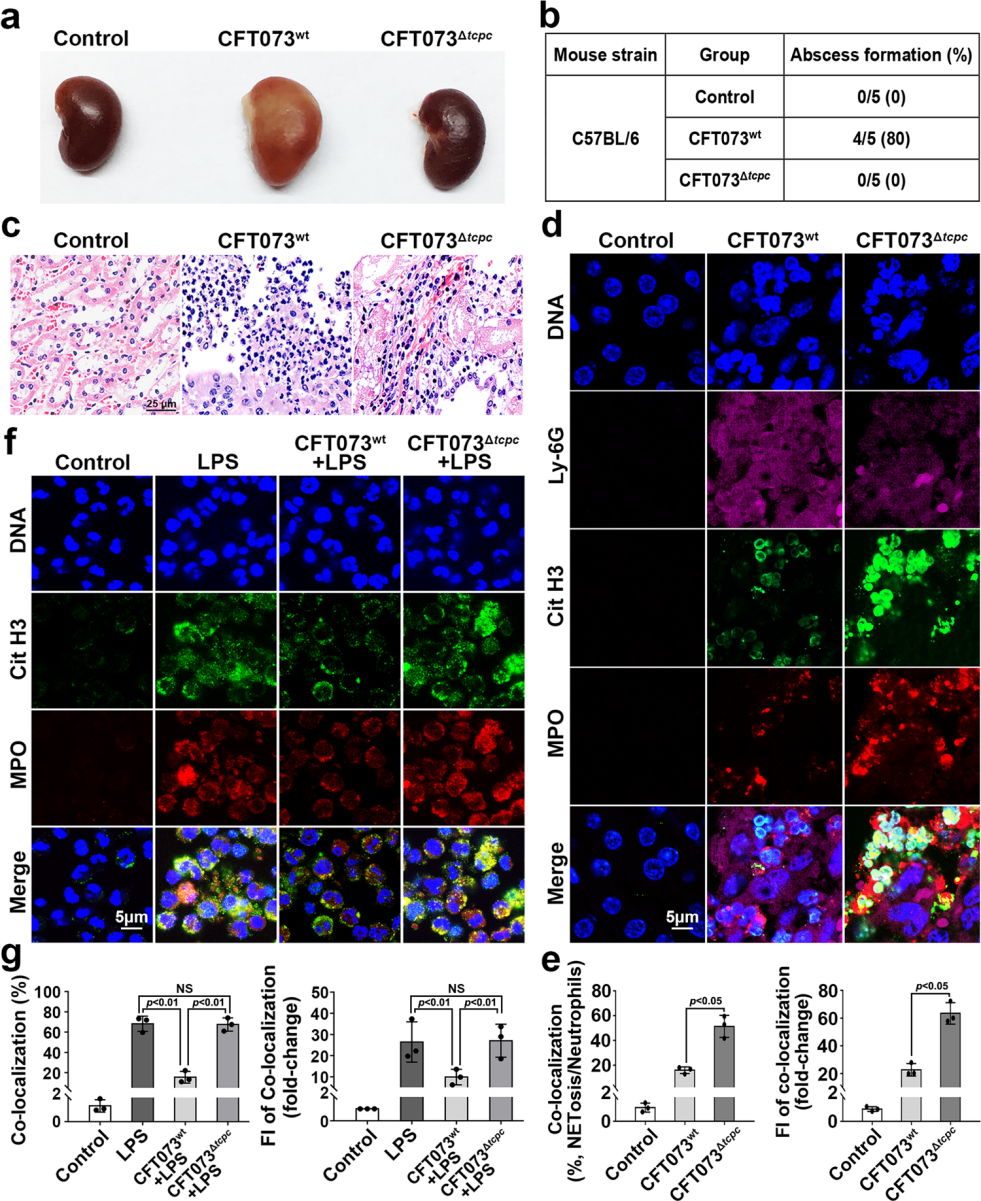
NETosis is inhibited in Kidneys of CFT073^wt^ induced pyelonephritis murine model and in CFT073^wt^-treated neutrophils. **a** Gross pathological observation of kidneys from CFT073^wt^- or CFT073^Δ*tcpc*^- induced pyelonephritis mouse models. One representative kidney from five mice in each group was shown. *n* = 5. **b** Abscess formation of kidneys in mice with different treatment. **c** Histological examination of kidneys from CFT073^wt^- or CFT073^Δ*tcpc*^-induced pyelonephritis mouse models. Scale bar = 25 μm. One representative image of kidneys from five mice in each group was shown, *n* = 3. **d** Confocal microscopy to examine in situ NETosis in kidneys from CFT073^wt^- or CFT073^Δ*tcpc*^-induced pyelonephritis mouse models. Scale bar = 5 μm. **e** Percent and FI of co-localization reflecting the NETosis levels in experiments as described in **d**. Mean ± SD of data from five kidneys in each group were shown, *n* = 5. FI in the control group was set as 1.0, *p* < 0.05 and *p* < 0.01 were considered to be statistically significant and extremely significant respectively. NS not significant. **f** Examination of in vitro LPS-induced NETosis of CFT073^wt^ or CFT073^Δ*tcpc*^ treated neutrophils by confocal microscopy. Scale bar = 5 μm. Images are representative of neutrophils from three different donors, *n* = 3. **g** Percent and FI of co-localization reflecting the NETosis levels in experiments as described in **f**. Mean ± SD of three independent experiments were shown, *n* = 3. *p* < 0.05 and *p* < 0.01 were considered to be statistically significant and extremely significant respectively. NS not significant. *p*-values were derived by Dunnett and Mann–Whitney tests. Source data for panel **e**, **g** are provided in the separate Source Data file.

The PN mouse models were induced by urethral instillation of CFT073^wt^ or *tcpc*-knock out CFT073 mutant (CFT073^Δ*tcpc*^) as described previously^5,21,22^. Abscesses in kidneys could be observed in CFT073^wt^-induced PN mouse model, no abscess was present in kidneys from CFT073^Δ*tcpc*^-infected mice (Fig. 1a, b). Accordingly, significantly increased infiltrates of neutrophil were observed in kidneys from the CFT073^wt^ group compared with those in kidneys from the CFT073^Δ*tcpc*^ group (Fig. 1c). These data demonstrate again that TcpC is a crucial virulence factor of UPEC. In situ NETosis in kidneys from PN mouse model induced by CFT073^wt^ was significantly inhibited when compared with that in kidneys from the CFT073^Δ*tcpc*^ infected group (Fig. 1d, e). To further confirm that this inhibition of in situ NETosis was caused by CFT073^wt^, human neutrophils were separately co-cultured in transwell with CFT073^wt^ or CFT073^Δ*tcpc*^ at a multiplicity of infection (MOI) = 100 for 12 h. 4 h before the end of the co-culture, 1 μg/ml LPS was added into the corresponding wells and NETosis was examined. NETosis in the CFT073^wt^ + LPS group was significantly inhibited compared with the LPS or CFT073^Δ*tcpc*^ + LPS groups. No significant inhibition of NETosis was observed in the group of CFT073^Δ*tcpc*^ + LPS compared with the LPS treatment group (Fig. 1f, g). These data demonstrate that CFT073^wt^ inhibits NETosis.

### Recombinant TcpC protein inhibits NETosis

**Fig. 2:**
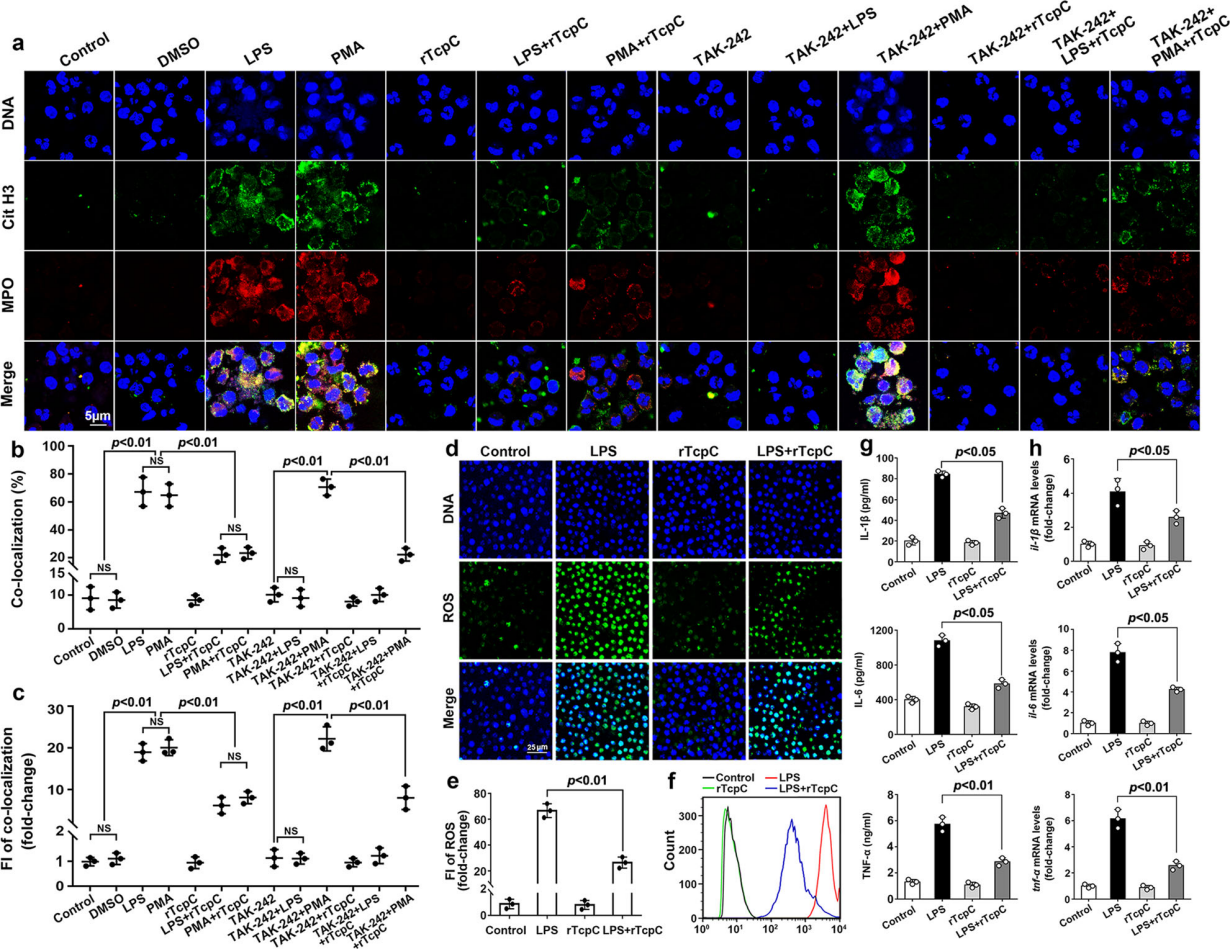
rTcpC inhibits NETosis. **a** Confocal microscopy to detect the influence of rTcpC on ROS-dependent NETosis. Scale bar = 5 μm. **b** Percent of co-localization reflecting the NETosis levels in experiments as described in **a**. Mean ± SD of three independent experiments were shown. **c** FI of co-localization in experiments as described in **a**. **b**, **c** *p* < 0.01 was considered to be extremely significant. The FI reflecting NETosis level in the control group was set as 1.0. Mean ± SD of three independent experiments were shown. *p* < 0.01 was considered to be extremely significant. NS not significant. *p*-values were derived by Mann–Whitney multiple comparisons test. **d** Confocal analyses of the influence of rTcpC on ROS production. Scale bar = 25 μm. Images shown are representative of three independent experiments. *n* = 3. **e** FI of ROS were analyzed by ImageJ software. Mean ± SD of three independent experiments were shown. *p* < 0.01 was considered to be extremely significant. *p*-values were derived by Dunnett comparison test. **f** Flow cytometry detection of the effects of rTcpC on ROS production in LPS-induced NETosis. **g**, **h** Protein and mRNA levels of IL-1β, IL-6, TNF-α were detected by ELISA and qRT-PCR, respectively. Mean ± SD of three independent experiments (*n* = 3). *p* < 0.05 and *p* < 0.01 were considered to be statistically significant and extremely significant respectively. *p*-values were derived by Dunnett comparison test. Source data for panel **b**, **c**, **e**, **g**, **h** are provided in the separate Source Data file.

Since CFT073^wt^ treatment caused inhibition of NETosis, recombinant TcpC protein (rTcpC) was prepared and its influence on NETosis was examined. The entry of rTcpC into neutrophils was verified by confocal and western blot analyses. rTcpC could enter the cells gradually with the treatment time (Supplementary Fig. 1a–c). Endocytosis is the process by which cells actively internalize molecules and surface proteins via an endocytic vesicle^23^. In order to confirm which endocytic vesicle mediated the entry of rTcpC, the cells were treated by clathrin-mediated endocytosis inhibitor dynasore^24,25^ and caveolin-dependent endocytosis inhibitor Methyl-β-cyclodextrin (MCD)^24,26^ for 30 min before treatment with rTcpC respectively. MCD but not dynasore blocked the entry of rTcpC into neutrophils, demonstrating that rTcpC could get into neutrophils through caveolin-mediated endocytosis (Supplementary Fig. 1d–f). LPS and PMA-induced ROS-dependent NETs formation, which is in line with previous report^27^. Although TAK-242, a TLR4 inhibitor^28^, did not influence PMA-induced NETosis, it completely blocked the LPS-induced NETosis. In the presence of rTcpC, both the LPS- and PMA-induced NETosis were significantly suppressed (Fig. 2a–c). In ROS-dependent NETosis, oxidative stress occurs in neutrophils. NADPH oxidase catalyzes the intracellular O_2_ to generate ROS that destroy pathogenic bacteria and promote translocation of MPO and NE to the nucleus, where NE proteolytically processes histones to disrupt chromatin packaging and MPO binds decondensing chromatin^29^, meanwhile expression of IL-1β, IL-6, TNF-α, and other proinflammatory factors is enhanced to achieve antibacterial effect^30,31^. The influence of rTcpC on ROS and proinflammatory cytokine production was examined. In the presence of rTcpC, LPS-induced production of ROS was profoundly inhibited while rTcpC itself did not affect the generation of ROS (Fig. 2d–f). Upon stimulation of LPS, enhanced expression of IL-1β, IL-6, and TNF-α occurred in neutrophils. However, rTcpC substantially inhibited this LPS-induced production of proinflammatory cytokines at both protein and mRNA levels (Fig. 2g, h). Moreover, when neutrophils were treated by CFT073^wt^, results with same trend were also obtained (Supplementary Fig. 2a, b). These data suggest that TcpC inhibits ROS-dependent NETosis.

### rTcpc not only inhibits the citrullination of chromatin histone, but also affects the transcription of related genes in nucleus

**Fig. 3:**
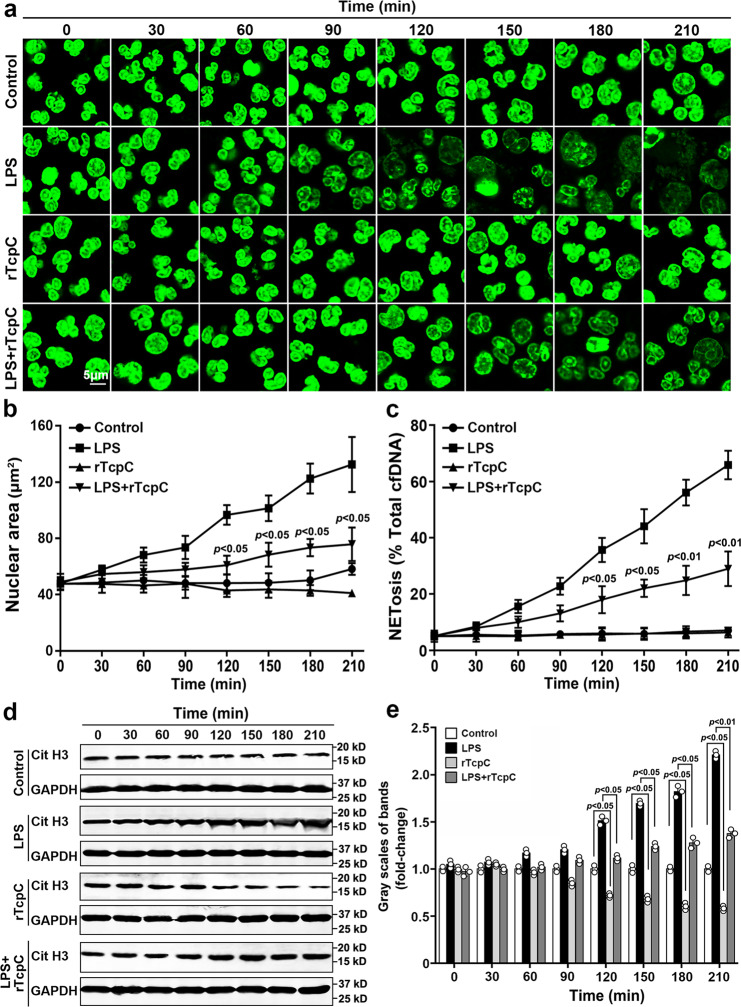
rTcpc inhibits chromatin decondensation in NETosis. **a** Dynamic analyses of chromatin decondensation by Sytoxgreen staining. Scale bar = 5 μm. **b** ImageJ analyses of DNA fluorescence area from experiments as described in **a**. **a**, **b** Mean ± SD are derived from three independent experiments in which five independent areas per condition were measured. *p* < 0.05 compared with LPS group, *p*-values were derived by Dunnett test. **c** ImageJ analyses of confocal images from experiments as described in **a**. Mean ± SD of three independent experiments were shown. *p* < 0.05 and *p* < 0.01 were considered to be statistically significant and extremely significant respectively, *p*-values were derived by Dunnett and Mann–Whitney tests. **d** Dynamic analyses of CitH3 in neutrophils with different treatment by western blot. **e** Gray scale analyses of bands from experiment as described in **d**. Mean ± SD of three independent experiments were shown, *n* = 3. *p* < 0.05 and *p* < 0.01 were considered to be statistically significant and extremely significant respectively, *p*-values were derived by Dunnett and Mann–Whitney tests. All western blots in panels **d** are provided as uncropped blots in Gels and Blots of Source Data file. Source data for panel **b**, **c**, **e** are provided in the separate Source Data file.

**Fig. 4:**
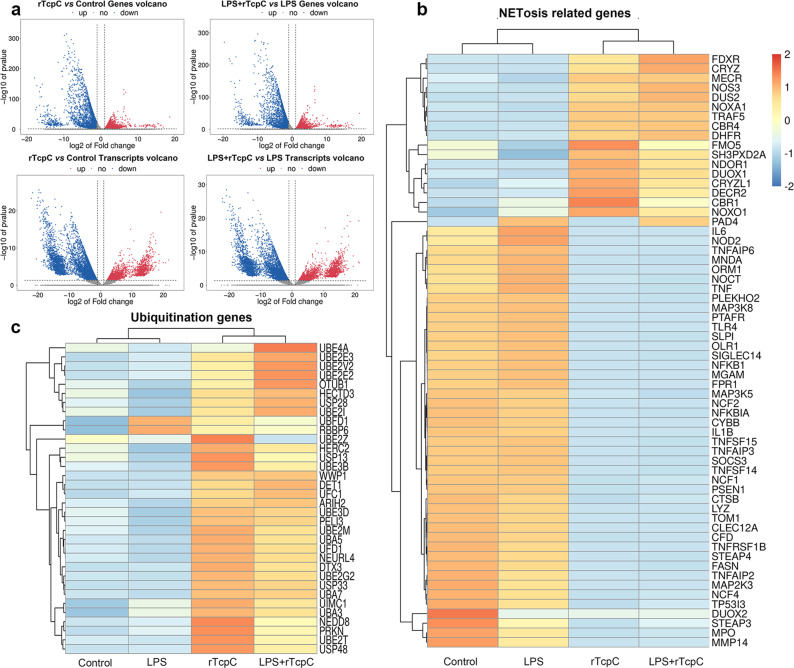
rTcpC affects the transcription of genes in neutrophils. **a** Genes and transcripts volcano in different treatment groups. **b** NETosis-related gene transcription levels in untreated (Control), LPS-, rTcpC-, or LPS + TcpC treated neutrophils. **c** Ubiquitination-related gene transcription levels in different treatment groups of neutrophils. **a**–**c** Data are representative of three biological repeats (*n* = 3). Source data for panel **a**–**c** are provided in the separate Source Data file.

During the ROS-dependent NETosis, NE digested histones are citrullinated by PAD4, resulting in chromatin decondensation^17,32^. Dynamic analyses of the influence of rTcpC on chromatin decondensation were carried out as described in previous reports^33,34^. Decondensation of chromatin increased along with the time in the LPS group. In the presence of rTcpC, however, the LPS-induced chromatin decondensation was significantly inhibited (Fig. 3a–c). Since CitH3 is also one of the indicators of chromatin decondensation^35^, CitH3 levels were dynamically analyzed. As shown in Fig. 3d, e, CitH3 levels also increased along with the time in LPS group, but this LPS-induced increase of CitH3 was suppressed significantly in the LPS + rTcpC group. Furthermore, rTcpC itself dynamically suppressed the levels of CitH3. Taken together, these data further indicate that rTcpC can inhibit chromatin depolymerization to form NETs. To determine the relevance of transcriptional activity during NETosis, a genome-wide transcriptomics analysis of neutrophils treated with LPS and/or rTcpC was conducted. rTcpC promoted gene transcripts down-regulation more than up-regulation (Fig. 4a). A large number of gene transcripts related to superoxidation and cytokines of ROS-dependent NETosis were inhibited (Fig. 4b), but ubiquitination-related gene transcripts were significantly up-regulated in rTcpC-treated neutrophils (Fig. 4c). Therefore, TcpC not only significantly inhibits the chromatin depolymerization, but also affects gene transcription during NETosis.

### rTcpC dose-dependently and dynamically decreases protein but not mRNA levels of PAD4 in neutrophils

**Fig. 5:**
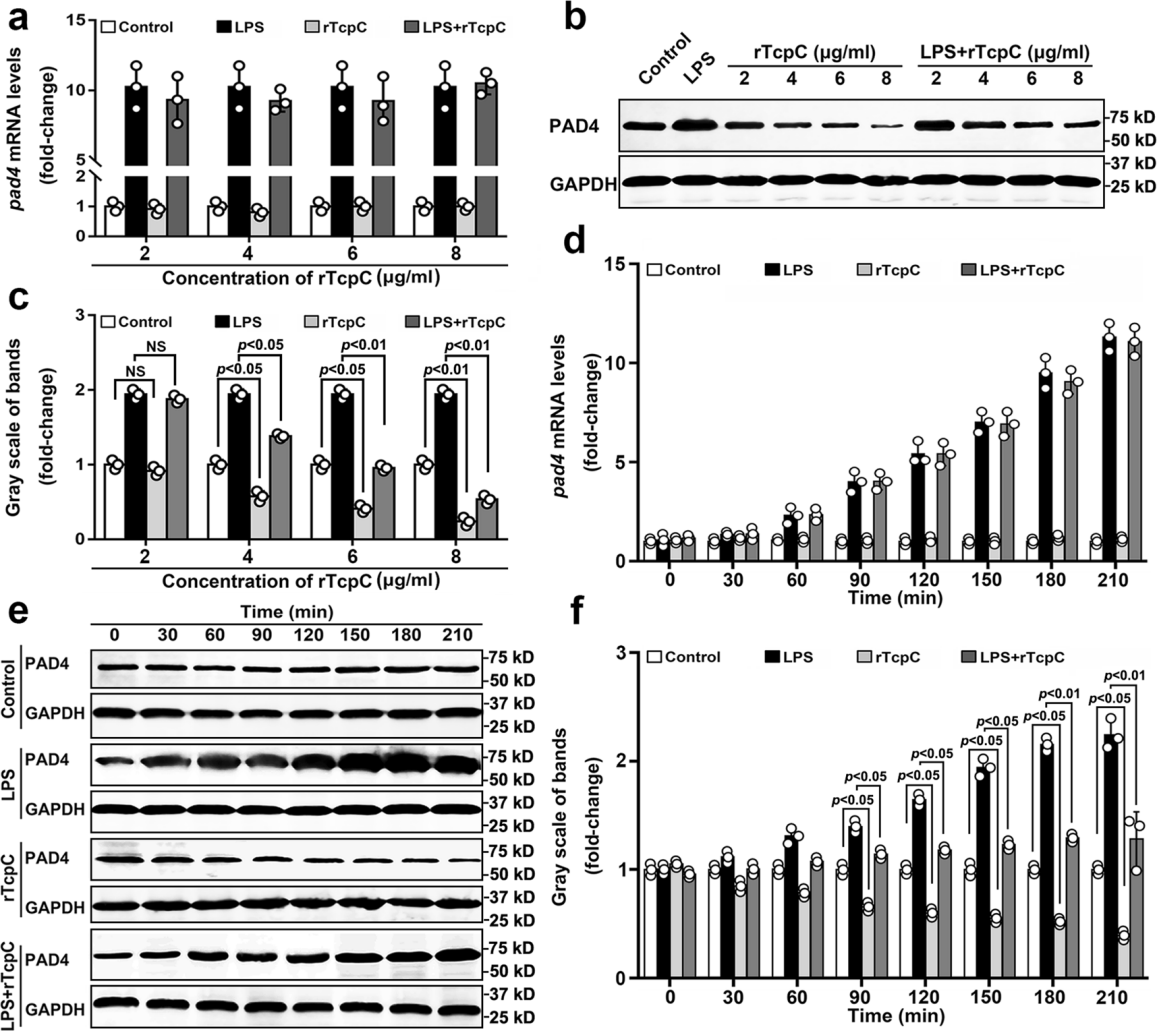
rTcpC inhibits protein but not mRNA levels of PAD4 in LPS-treated neutrophils. **a** qRT-PCR to examine mRNA levels of PAD4 in different treatment groups of neutrophils. Data are from three independent experiments (*n* = 3) with three replicates per condition. **b** Western blot analyses of PAD4 protein levels in neutrophils with different treatment. **c** Gray scale analyses of bands from experiments as described in **b**. Gray scale values reflecting the PAD4 levels in the control group were set as 1.0. Mean ± SD of three independent experiments were shown. *p* < 0.05 and *p* < 0.01 were considered to be statistically significant and extremely significant respectively, NS not significant. **d** Dynamic analyses of the influence of rTcpC on *pad4* mRNA levels in neutrophils with different treatment. The data of *pad4* mRNA levels are from 3 independent experiments (*n* = 3). **e** Dynamic analyses of PAD4 in neutrophils with different treatment by western blot. **f** Gray scale analyses of bands from experiment as described in **e**. Mean ± SD of three independent experiments were shown. *p*-values were derived by Dunnett and Mann–Whitney tests. All western blots in panels **b**, **e** are provided as uncropped blots in Gels and Blots of Source Data file. Source data for panel **a**, **c**, **d**, **f** are provided in the separate Source Data file.

Because PAD4-mediated histone citrullination is the essential step in NETs formation^9,13,36^, dose-dependent and dynamic analyses of the influence of rTcpC on PAD4 expression were examined. In accordance with the results of transcriptomics analyses (Fig. 4b), upon LPS stimulation, both mRNA and protein levels of PAD4 increased significantly (Fig. 5a–f). Although rTcpC treatment did not affect LPS-induced mRNA expression of PAD4 (Figs. 4b and 5a, d), rTcpC itself could dose-dependently inhibit protein levels of PAD4 compared with the control group (Fig. 5b, c). Importantly, dose-dependent and dynamical inhibition of PAD4 protein levels were observed in the LPS + rTcpC group when compared with the LPS group (Fig. 5b, c and e, f). Furthermore, when neutrophils were treated with CFT073^wt^ and CFT073^Δ*tcpc*^, similar results were obtained (Supplementary Fig. 3a, b) These data show that TcpC decreases protein but not mRNA levels of PAD4 in neutrophils.

### rTcpC is an E3 ubiquitin ligase that promotes ubiquitination of PAD4

**Fig. 6:**
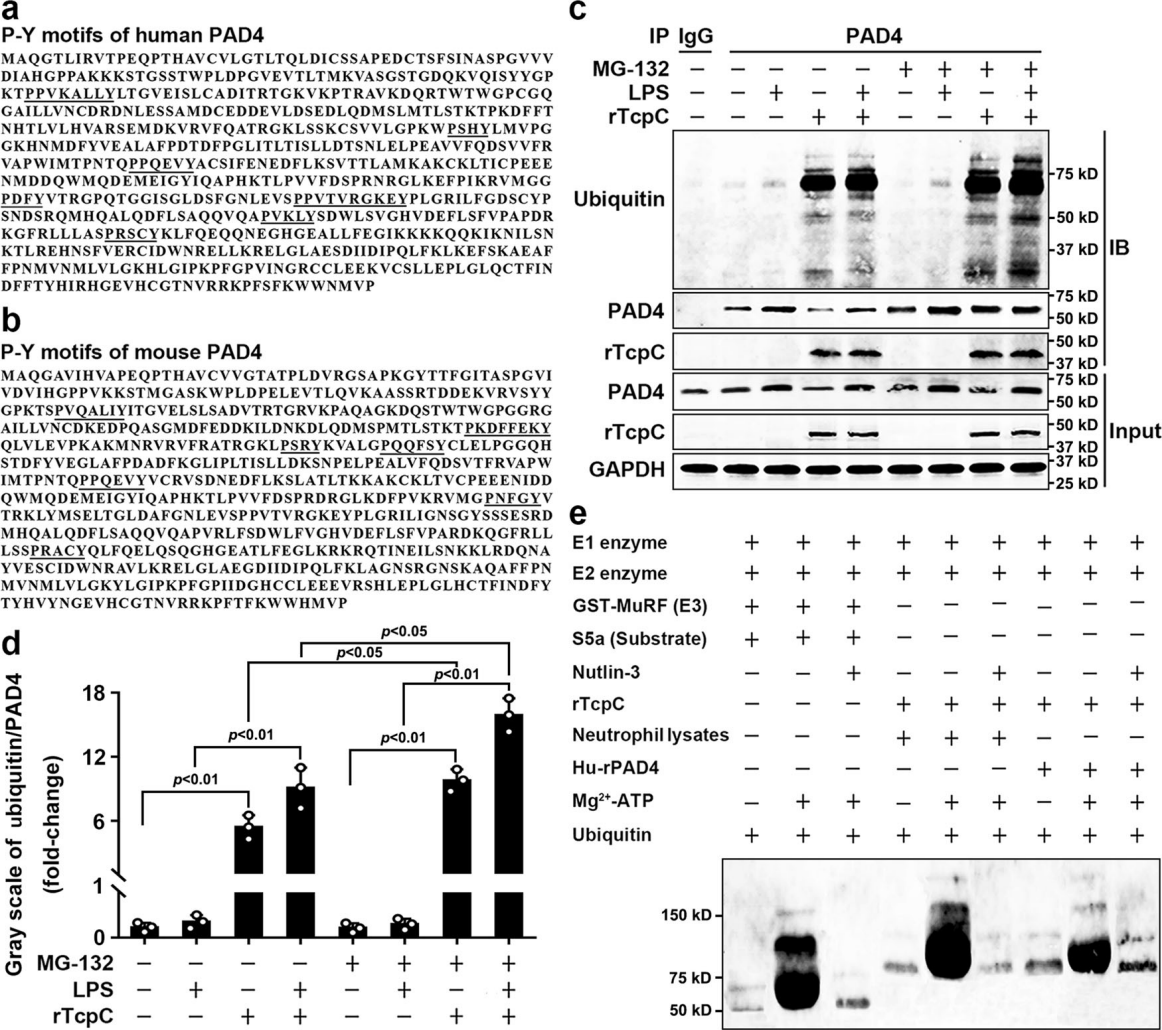
TcpC is an E3 ligase that promotes ubiquitination of PAD4. **a**, **b** Bioinformatics prediction of ubiquitination functional sites in amino acid sequences of human PAD4 (NCBI Reference Sequence: BAA84542.1) and mouse PAD4 (NCBI Reference Sequence: NP_035191.2). **c** Co-immunoprecipitation and immunoblotting analyses of ubiquitination of PAD4 in human neutrophils. Western blot data are representative of three independent experiments, *n* = 3. **d** Gray scale analyses of the ubiquitin/PAD4 from experiments as described in **c**. Mean ± SD of three independent experiments were shown. *p* < 0.05 and *p* < 0.01 were considered to be statistically significant and extremely significant respectively. **e** In vitro ubiquitination kit to examine the PAD4-targeted E3 activity of rTcpC, western blot data is representative of three independent experiments, *n* = 3. *p*-values were derived by Dunnett and Mann–Whitney multiple comparison tests. All western blots in panels **c**, **e** are provided as uncropped blots in Gels and Blots of Source Data file. Source data for panel **d** are provided in the separate Source Data file.

Ubiquitination is an important protein post-translational modification pathway in eukaryotic cells^37^. Ubiquitination is mediated by ubiquitin-activating (E1), ubiquitin-conjugating (E2), and ubiquitin-ligating (E3) enzyme cascade reactions^38^. In the process of ubiquitination, E3 binds not only to E2, but also specifically to substrate proteins^39^. In human genome, >600 annotated E3 ligases have been found, they are divided into three types of domains: RING, HETC, and RBR^40^. In the process of ubiquitination by HETC E3 ligase, the conserved cysteine (Cys) residue at N-terminus of the enzyme is necessary^37,41^. HECT E3 ligase contains tryptophan-tryptophan (WW) domains that bind to proline-tyrosine (P-Y) motif containing substrates^42–44^. In our previous studies, we have demonstrated for the first time that TcpC is a HECT family E3 ubiquitin ligase that can promote ubiquitination of MyD88^45^. To examine whether TcpC can also serve as the E3 that promote PAD4 ubiquitination, we did bioinformatics analyses at first and found that human and mouse PAD4 contain P-Y motifs (Fig. 6a, b), suggesting that PAD4 is probably one of the TcpC E3 enzyme substrates. Then, the influence of rTcpC on ubiquitination of PAD4 was examined by co-immunoprecipitation and immunoblotting assays. Although rTcpC treatment decreased the PAD4 protein level in lysates of neutrophils, the ubiquitination of PAD4 was significantly enhanced compared with the LPS-treated group. When the proteasome activity was blocked by MG-132, the ubiquitination of PAD4 was enhanced further in the rTcpC-treated groups (Fig. 6c, d). These data demonstrate that rTcpC promotes ubiquitination of PAD4. At last, the PAD4-targeted E3 activity of rTcpC was further examined by in vitro ubiquitination kit analyses which mimic the ubiquitination process as previously described^46,47^. In these assays, the GST-MuRF (E3) and its substrate S5a were served as the positive control. When the E3 in the kit was replaced by rTcpC and lysates of neutrophils and commercially available human recombinant PAD4 (Hu-rPAD4) used as the substrates, substantially enhanced ubiquitination of PAD4 could be observed. Furthermore, this rTcpC mediated enhanced ubiquitination of PAD4 could be abrogated when the E3 inhibitor Nutlin-3 was employed (Fig. 6e). These data firmly demonstrate that TcpC is an E3 ubiquitin ligase which promotes ubiquitination of PAD4.

### TcpC promotes accumulation of PAD4 in proteasomes

**Fig. 7:**
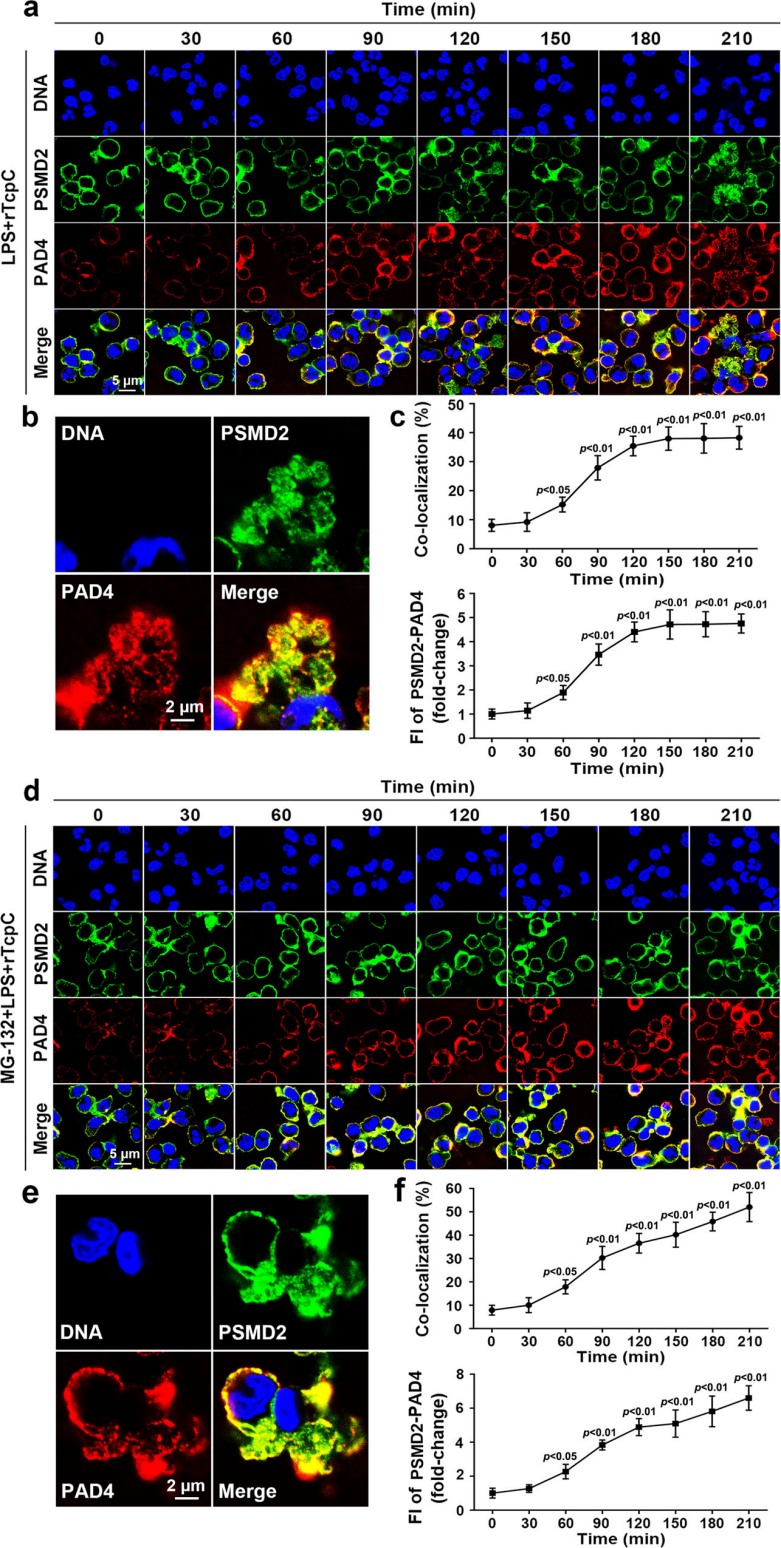
rTcpC treatment leads to accumulation of PAD4 in proteasomes. **a** Dynamic observation of co-localization of PAD4 with PSMD2 by confocal microscopy. Scale bar = 5 μm. **b** Enlarged image of the co-localization in **a** at 210 min. Scale bar = 2 μm. Confocal microscopy images are representative of three independent experiments from three donors’ neutrophils, *n* = 3. **c** Percent and FI of co-localization were analyzed by ImageJ software. Mean ± SD of three independent experiments as described in **a** were shown. *p* < 0.05 and *p* < 0.01 compared with the group at 0 min. **d** Dynamic observation of co-localization of PAD4 with PSMD2 in the presence of MG-132 by confocal microscopy. Scale bar = 5 μm. **e** Enlarged image of the co-localization in **d** at 210 min. Scale bar = 2 μm. Confocal microscopy images are representative of three independent experiments from three donors’ neutrophils, *n* = 3. **f** Percent and FI of co-localization were analyzed by ImageJ software. Mean ± SD of three independent experiments as described in **d** were shown. *p* < 0.05 and *p* < 0.01 compared with the group at 0 min. *p*-values were derived by Dunnett test. Source data for panel **c** and **f** are provided in the separate Source Data file.

In the process of ubiquitination, E3 ubiquitin ligase transports substrate protein to proteasome for degradation^48,49^. In order to verify if rTcpC treatment leads to accumulation of PAD4 in the proteasomes, confocal microscopy was employed to examine co-localization of PAD4 with the proteasome marker, non-ATPase 2 (PSMD2)^50,51^. Dynamic analyses of PAD4 accumulation in proteasomes showed that, in the absence of MG-132, co-localization of PAD4 and PSMD2 increased 60 min later, and reached a plateau 120 min after rTcpC treatment (Fig. 7a–c). In the presence of MG-132, however, co-localization of PAD4 with PSMD2 increased steadily after 60 min treatment with rTcpC (Fig. 7d–f). These data suggest that rTcpC promotes PAD4 accumulation in proteasomes in LPS-stimulated neutrophils.

### C12S point mutant rTcpC-C12S loses the ability to inhibit NETosis

**Fig. 8:**
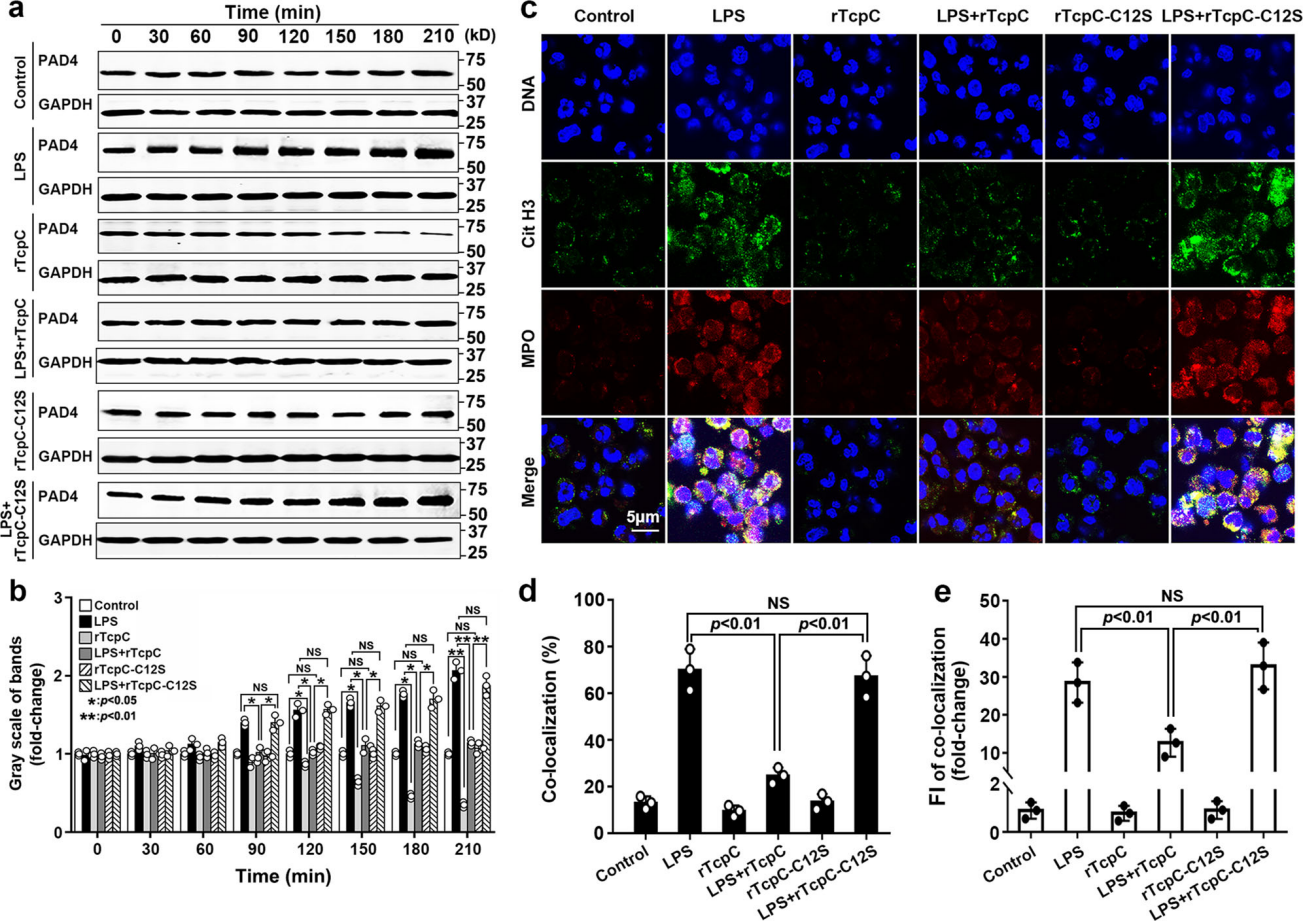
rTcpC-C12S loses ability to promote degradation of PAD4 and inhibit LPS-induced NETosis. **a** Western blot analyses to detect the ability of rTcpC-C12S to promote rPAD4 degradation. One representative of three independent experiments was shown, *n* = 3. **b** Gray scale analyses of bands from experiments as described in **a**. Gray scale values reflecting the PAD4 levels in the control group were set as 1.0. Mean ± SD of three independent experiments were shown. *p* < 0.05 and *p* < 0.01 were considered to be statistically significant and extremely significant respectively, NS not significant. **c** Confocal microscopy to detect the ability of rTcpC-C12S to inhibit LPS-induced NETosis. Scale bar = 5 μm. **d** Percent of co-localization was analyzed by ImageJ software. Mean ± SD of three independent experiments as described in **c**, *p* < 0.01 was considered to extremely significant. **e** FI of co-localization was analyzed by ImageJ software. FI in control group was set as 1.0. Mean ± SD of three independent experiments as described in **c** were shown, *n* = 3, *p* < 0.01 was extremely significant. All western blots in panels **a** are provided as uncropped blots in Gels and Blots of Source Data file. *p*-values were derived by Dunnett and Mann–Whitney multiple comparison tests. Source data for panel **b**, **d** and **e** are provided in the separate Source Data file.

In our previous studies, we have demonstrated that C12 is the key amino acid to retain the E3 activity of TcpC^45^, the influence of C12S point mutant rTcpC-C12S on degradation of PAD4 and LPS-induced NETosis was examined. Compared with the rTcpC prototype, rTcpC-C12S showed significantly attenuated activity to promote degradation of PAD4 (Fig. 8a, b) and to inhibit LPS-induced NETosis (Fig. 8c–e), suggesting further that the inhibitory effect of TcpC on NETosis is mediated by promoting degradation of PAD4.

## Discussion

Although the pathophysiological roles of NETosis have not been fully understood, NETosis is an important anti-infection mechanism of neutrophils. In ROS-dependent NETosis, NADPH oxidase complex catalyzes O_2_ to produce ROS which activate transcription and activation of downstream NETosis essential enzyme genes^52^. Among them, PAD4 is of the most important. The conversion of histone arginine into citrulline catalyzed by PAD4, which reduces the positive charges of histone and causes chromatin entropic swelling, is the most important event that drives the release of NETs^9^. After chromatin entropic swelling the process of NET formation cannot be reversed. Therefore, histone citrullination can be regarded as the “turning-point” in NETosis. Although the exact signaling pathway that regulates PAD4 expression has not been clarified, manipulating PAD4 to inhibit NETosis might be a vital strategy for pathogens to evade effector function of neutrophils. On the one hand, it was found that TcpC impedes TLR signaling pathway, hereby inhibiting macrophage-mediated innate immunity^5,21^. On the other hand, TcpC promotes kidney cells to produce CXCL2 chemokine which attracts neutrophil infiltration in kidney, the characteristic pathological changes of acute PN^22^. But the influence of TcpC on NETosis has been remained elusive.

In the present study, we show that in situ NETosis in kidneys of PN murine model induced by CFT073^wt^ was significantly inhibited compared with that in CFT073^Δ*tcpc*^ infected mouse (Fig. 1d, e). In line with the results of in situ NETosis, LPS-induced in vitro NETosis was also suppressed significantly in CFT073^wt^- (Fig. 1f, g) or rTcpC-treated (Fig. 2a–c) human neutrophils. These data demonstrate that TcpC suppresses NETosis.

During ROS-dependent NETosis, ROS is produced to kill pathogens and stimulate NE to digest histone for citrullination by PAD4 in nucleus^20,53^. Furthermore, proinflammatory cytokines such as IL-1β, IL-6, TNF-α are up-regulated when neutrophils undergo NETosis^54^. In order to explore the mechanisms underlying TcpC-mediated inhibition of NETosis, we examined the influence of TcpC on expression of ROS and proinflammatory cytokines as well as PAD4. We found that rTcpC profoundly inhibited LPS-induced production of ROS (Fig. 2d–f) and expression of proinflammatory cytokines at both protein and mRNA levels (Fig. 2g, h) in neutrophils. But unlike the influence on proinflammatory cytokines, rTcpC suppressed, in a dose- and time-dependent manner, protein (Fig. 5b, c, e, f) but not mRNA (Fig. 5a, d) levels of PAD4, suggesting that rTcpC decreases PAD4 protein by post-transcriptional modification. Because level of CitH3 is determined by PAD4 and rTcpC decreases concentration of PAD4 in neutrophils, it is no wonder that rTcpC itself can dynamically suppress levels of CitH3 (Fig. 3d).

It has been demonstrated that ubiquitination plays an important role in protein post-translational modification in eukaryotic cells that regulates many biological processes^39,48,55–57^, and in many aspects of the immune system, including innate and adaptive immunity and antimicrobial autophagy. In addition, increasing evidence indicates that microbial pathogens exploit the ubiquitin pathway to evade the host immune system^58,59^. Furthermore, our previous studies have demonstrated that TcpC is an E3 ubiquitin ligase of HECT family^45^, the influence of rTcpC on ubiquitination of PAD4 was examined. Our data clearly show that rTcpC treatment caused significantly enhanced ubiquitination of PAD4 in neutrophils (Fig. 6c, d). Bioinformatics analyses showed that in the amino acid sequence of human (Fig. 6a) and mouse (Fig. 6b) PAD4 there are P-Y motifs of the E3 ligase substrates, suggesting that TcpC might be a PAD4-targetd E3 ubiquitin ligase. This speculation was further verified by in vitro ubiquitination kit assays. rTcpC could serve as the E3 to promote ubiquitination of both proteins in the lysate of neutrophils and Hu-rPAD4, and this E3 activity of rTcpC could be abrogated by the E3 inhibitor Nutlin-3 (Fig. 6e). These data confirm that TcpC is a PAD4-targeted E3 ubiquitin ligase.

To examine whether rTcpC treatment promotes accumulation of PAD4 in proteasome. Co-localization of PAD4 with the proteasome maker PSMD2 in neutrophils was observed by confocal microscopy. rTcpC treatment led to increased accumulation of PAD4 in proteasomes in LPS-treated neutrophils (Fig. 7a–c). Particularly, when the proteasome activity was blocked by MG-132, accumulation of PAD4 in proteasome increased steadily during the whole treatment period (Fig. 7d–f). These data confirm that TcpC promotes degradation of PAD4 through ubiquitin-proteasome pathway.

At last, we examined the influence of rTcpC-C12S on degradation of PAD4 and LPS-induced NETosis. In line with the changes of activity in degrading PAD4 (Fig. 8a, b), rTcpC-C12S showed significantly decreased activity to inhibit the LPS-induced NETosis compared with the rTcpC prototype (Fig. 8c–e). These data suggest that the inhibitory effect of TcpC on NETosis is primarily mediated by promoting ubiquitinated degradation of PAD4.

**Fig. 9:**
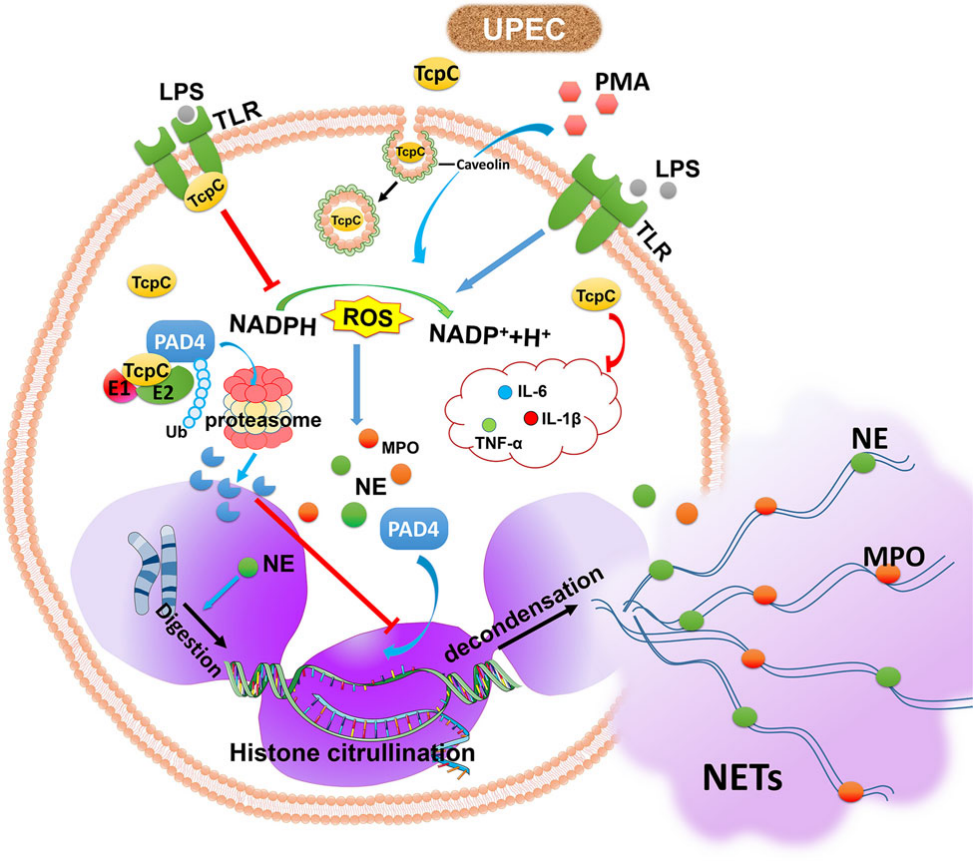
Schematic diagram of the mechanisms underlying TcpC-mediated inhibition of NETosis. Upon stimulation of LPS and PMA, NADPH oxidase catalyzes the intracellular O_2_ to generate ROS. ROS activate downstream NETosis essential enzyme, such as MPO, NE, and PAD4. NEs digest nucleosomal histones and promote chromatin relaxation, which favors PAD4 to catalyze histone into citrullinated histone. Citrullination of histones causes chromatin entropic swelling and release of NETs. In case of TcpC-secreting UPEC infection, TcpC inhibits pathogen-induced NETosis via two mechanisms: (1) Inhibition of ROS production, and (2) Functioning as an E3 ubiquitin ligase that promotes PAD4 degradation through ubiquitin-proteasome pathway.

In summary, this study demonstrated that TcpC inhibits NETosis by serving as a PAD4-targeted E3 ubiquitin ligase that promote degradation of PAD4 via ubiquitin-proteasome pathway (Fig. 9). Our findings provide not only a novel mechanism by which TcpC-secreting UPECs evade host innate immune response, but also new clues to clarify the pathogenicity of other microbial pathogens.

## Methods

### Ethics statement

Animal experiments were performed in accordance with the National Regulations for the Administration of Experimental Animals of China (1988–002) and the National Guidelines for Experimental Animal Welfare of China (2006–398). All the animal experimental protocols were approved by the Ethics Committee for Animal Experiment of Zhejiang University City College School of Medicine and fully informed consent of all healthy blood donors was obtained after clearing the possible consequences of the study.

### Reagents

Fetal bovine serum (FBS, Gibco, USA, 10100147), RPMI 1640 medium (Gibco, USA, 22400105), LPS (Sigma, USA, L2630), Sytox Green (Invitrogen, USA, S7020), IL-1β, IL-6, TNF-α ELISA Kit (eBioscience, USA, KAC1211, EH2IL6, KHC3011), DCFH-DA probe dye (Thermo, USA, I36007), BCA Protein Concentration Kit (Beyotime, China, P0012S), rabbit anti-PAD4-IgG (Abcam, UK, ab96758), rabbit anti-MPO-IgG (Abcam, UK, ab208670), rabbit anti-ubiquitin-IgG (Abcam, UK, ab134953), anti-GAPDH-IgG (Abcam, UK, ab181602), Anti-PSMD2-IgG (Abcam, UK, ab125914), Alexa fluor 647 nm goat anti-rabbit IgG (Invitrogen, USA, A-21244) and Alexa fluor 488 nm goat anti-mouse IgG fluorescent secondary antibody (Invitrogen, USA, A-11001), DAPI (Invitrogen, USA, D1306), Ly-6G/Ly-6C (PE, Thermo, USA, 14-5931-81), CD11b (FITC, Thermo, USA, 11-0112-41), Nutlin-3 (Selleckchem, USA, S1061), MG-132 (Selleckchem, USA, S2619), primer (Invitrogen, USA), RNAiso Plus (TaKaRa, Japan, 9109), PrimeScript^TM^ RT reagent Kit with gDNA Eraser (TaKaRa, Japan, RR047A), TB Green TM Premix Ex Taq^TM^ (TaKaRa, Japan, RR071A), Pierce^TM^ Protein A/G magnetic beads (Thermo, USA, 88802), MuRF1 Ubiquitin Ligase Kit-S5a Substrate (Boston Biochem, USA, K-102), rabbit anti-Ly-6G-IgG (Abcam, UK, ab238132), Alexa fluor 546 nm goat anti-rabbit IgG (Thermo, USA, A-11035), rabbit anti-CitH3-IgG (Abcam, UK, ab5103), Alexa fluor 488 nm goat anti-rabbit IgG fluorescent secondary antibody (Invitrogen, USA, A-11008), ProLong Diamond Antifade Mountant (Thermo, USA, P36990), Dynasore (MCE, USA, HY-15304), TAK-242 (MCE, USA, HY-11109), Methyl-β-cyclodextrin (MCE, USA, HY-101461), and recombinant TcpC protein and rabbit anti-TcpC-IgG (made in our laboratory).

### Mouse PN models and in situ NETosis examination

Female C57BL/6 mice, 6–8 weeks of age, were provided by Shanghai Slack Laboratory Animals Co., Ltd (Shanghai, China). PN murine models were induced by transurethral instillation of CFT073^wt^ or CFT073^Δ*tcpc*^ as described in our previous report^22^. Normal saline-treated mice served as the control group. In all, 72 h later, the model mice were sacrificed and kidneys were removed for pathological and in situ NETosis examinations as described previously^60^. After the sections were fixed and tissue antigen repairing, the fixed tissues were incubated with rabbit anti-Ly-6G-IgG, rabbit anti-CitH3-IgG, and rabbit anti-MPO-IgG (Abcam, UK, 1:200 dilution) overnight at 4 °C and stained with different fluorescent secondary antibody (Alexa fluor 546 nm goat anti-rabbit IgG, Alexa fluor 488 nm goat anti-rabbit IgG and Alexa fluor 647 nm goat anti-rabbit IgG, 1:500 dilution) in dark respectively, then added ProLong Diamond Antifade Mountant (Thermo, USA) and DAPI to each coverslip, the coverslips were placed on the slides and confocal microscope images were taken.

### Histological examination

Kidneys were fixed in freshly prepared 4% paraformaldehyde soon after dissection and incubated overnight at 4 °C. The fixed tissues were incubated in 15% sucrose at 4 °C and washed in 25% ice cold sucrose at 4 °C. Tissues were then frozen in isopentane at −80 °C. Cryostat sections were made, mounted onto poly-d-lysine-coated glass slides and stained with htx-eosin^21^.

### Isolation of human neutrophils

EDTA-anticoagulated peripheral blood from healthy donors was used to isolate neutrophils using EasySep Direct Human Neutrophil Isolation Kit (StemCell Technologies, USA) following the manufacturer’s instructions. Erythrocytes were lysed with red blood cell lysis buffer and washed with PBS. Subsequently, purity of the human neutrophils was evaluated by FACS using antibodies against Ly-6G/Ly-6C (1:500 dilution, Ly-6G/Ly-6C monoclonal antibody, PE, Thermo, USA) and CD11b (1:500 dilution, CD11b monoclonal antibody, FITC, Thermo, USA). Neutrophil purity was >93% (Supplementary Fig. 4).

### Transwell co‑culture

Neutrophils (1 × 10^6^) were separately co-cultured for 12 h, in transwell (Corning, USA) at a multiplicity of infection (MOI) = 100, with CFT073^wt^ or CFT073^*Δtcpc*^. Neutrophil cultures without *E. coli* treatment served as the control group. In all, 4 h before the end of the culture, 1 μg/ml LPS was added into corresponding wells. Then the cells were harvested and subjected to confocal microscopy to examine NETosis.

### rTcpC treatment of neutrophils

In all, 1 × 10^6^ neutrophils were treated, in the presence or absence of 1 μg/ml LPS, 100 nM PMA, with or without 20 μM TAK-242, 4 μg/ml rTcpC for 210 min. Neutrophils cultured in medium alone was set as control group. Culture supernatants were collected for measurement of proinflammatory cytokines and cells were subjected to detection of NETosis, qRT-PCR analyses of cytokine and *pad4* mRNA and western blot analyses of PAD4, respectively.

### Confocal microscopy to examine NETosis

The above treated neutrophils were fixed by 4% paraformaldehyde in PBS overnight at 4 °C, then permeabilized with 0.1% TritonX-100 in PBS for 20 min, and washed three times with PBS. Cells were blocked with 1% BSA, 0.1% Tween 20 in PBS for 2 h at room temperature, and then incubated with rabbit anti-MPO antibodies (Abcam, UK, 1:200 diluted in 0.1% Tween 20 in PBS) overnight at 4 °C. Next, neutrophils were gently washed three times with PBS and incubated in the dark with goat anti-rabbit IgG antibody (Alex Flour 647 nm, 1:500 diluted in the PBS) for 2 h at room temperature. Before staining with 100 ng/ml DAPI, neutrophils were incubated with rabbit anti-CitH3 antibodies (Abcam, UK, 1:200 diluted in 0.1% Tween 20 in PBS) overnight at 4 °C, then washed three times and incubated with Alexa fluor 488 nm goat anti-rabbit IgG fluorescent secondary antibody, finally washed with PBS and ProLong Diamond Antifade Mountant (Thermo, USA) was added to each coverslip, the coverslips were placed on the slides, and then observed with a confocal fluoresce microscope (FV3000 Olympus, Japan). Images were analyzed with the ImageJ software from National Institutes of Health^33,61^.

### Quantification of NETs

NETs formation was quantified by detecting DNA release spectrophotometrically with the DNA-binding dye Sytox Green as previously described^33^. Briefly, neutrophils were treated for 210 min with or without 4 μg/ml rTcpC in the presence or absence of 1 μg/ml LPS, at the beginning of the incubation 500 nM Sytox Green were added into each well. Fluorescence values were measured every 30 min with the 488 nm excitation. Scale was set and threshold adjusted to define the area of fluorescent DNA. Total area of all fluorescent particles indicated the amount of NETs formation. Unstimulated neutrophils had an area of 62 ± 6.1 µm^2^. Thus only particles >70 µm^2^ were considered NETs.

### Confocal microscopy to detect ROS production

In all, 1 × 10^6^/ml neutrophils were treated with or without 4 μg/ml TcpC and 1 μg/ml LPS for 210 min. Cells were harvested and resuspended in 1 ml serum-free culture medium. Cells were stained with 1 μl DCFH-DA probe dye (Invitrogen, USA) and 100 ng DAPI (Invitrogen, USA) in dark for 20 min at 37 °C. After three times wash with PBS, cells in each group were adjusted to a density of 1 × 10^6^ cells/ml. In total, 10 μl cells were put on a Poly-d-Lys (Sigma, USA) pre-coated coverslip. In all, 1 min later the coverslip was put on a slide and observed with a confocal microscope (FV3000 Olympus, Japan). Images were analyzed with the ImageJ software.

### ELISA

Concentrations of IL-1β, IL-6, and TNF-α in supernatants of neutrophils were measured by ELISA according to the instructions of the manufacturer (eBioscience, USA).

### qRT-PCR

Total RNA of treated neutrophils was extracted by RNAiso Plus (TaKaRa, Japan), and cDNA was synthesized by reverse transcription using PrimeScript^TM^ RT reagent kit with gDNA Eraser (TaKaRa, Japan). The mRNA levels of IL-1β, IL-6, TNF-α, and PAD4 in neutrophils were detected by qRT-PCR, and β-actin was set as an internal reference. The primers are listed in Table 1. The Cq value was calculated by 2^−ΔΔCT^ method.

**Table 1:** Information of primers used in this study.

Primer	Sequence (5′ to 3′)	Purpose
*pad4*	F: CTCTCCAGGAGTCATCGTAG	Detection of *pad4* mRNA
	R: CCAACACCAGCTGATACTTT	
*il-1β*	F: CCTTGTGCAAGTGTCTGAAG	Detection of *il-1β* mRNA
	R: GGGCTTGGAAGCAATCCTTA	
*il-6*	F: GCCCTTCAGGAACAGCTATGA	Detection of *il-6* mRNA
	R: TGTCAACAACATCAGTCCCAAGA	
*tnf-α*	F: CCTGTAGCCCACGTCGTAG	Detection of *tnf-α* mRNA
	R: GGGAGTAGACAAGGTACAACCC	
*β-actin*	F: ATGGATGACGATATCGCTG	Inner reference
	R: AACACCCATTCCCTTCACAG	

*F* forward primer, *R* reverse primer.

### Flow cytometry analyses of ROS

The ROS generation during NETosis was detected by FACS using DCFH-DA (Invitrogen, USA). Before stimulation, neutrophils were preloaded with DCFH-DA for 20 min, cells were washed three times with PBS. Then, neutrophils were treated with LPS or rTcpC for 210 min. Finally, the fluorescence signals of DCFH-DA were read at FITC channel on a BD FACSCalibur.

### Western blot analyses of PAD4

Proteins in supernatants of lysates from treated neutrophils were prepared. After 12% SDS-PAGE, proteins were electro-transferred onto PVDF membranes. Proteins were probed with rabbit anti-PAD4 IgG (1:10000 dilution, Abcam, UK) as primary antibody and IRDye® 680RD goat anti-rabbit-IgG (H + L) (1:5000 dilution, LI-COR, USA) as secondary antibody. Images were developed using ODYSSEY® CLx Infrared Imaging System (LI-COR). Gray scale values of bands were analyzed by ImageJ software.

### Dynamic analyses of CitH3 levels

Neutrophils (1 × 10^6^) were treated with or without 4 μg/ml rTcpC for the indicated time in the absence or presence of 1 μg/ml LPS. Proteins in supernatants of lysates from treated neutrophils were prepared. After 12% SDS-PAGE, proteins were electro-transferred onto PVDF membranes. Proteins were probed with rabbit anti-CitH3 IgG (1:5000 dilution, Abcam, UK) as primary antibody and IRDye® 680RD goat anti-rabbit-IgG (H + L) (1:5000 dilution, LI-COR, USA) as secondary antibody. Images were developed using ODYSSEY® CLx Infrared Imaging System (LI-COR). Gray scale values of bands were analyzed by ImageJ software.

### Co-immunoprecipitation and immunoblotting to detect ubiquitination of PAD4

The treated neutrophils were washed twice with PBS and were lysed with RIPA. After centrifugation at 12,000×*g* for 30 min at 4 °C, the supernatants were collected and protein concentrations were determined by a BCA Protein Assay Kit (Beyotime, China). Proteins in cell lysates were then immunoprecipitated with the rabbit anti-PAD4 IgG (1:1000 dilution, Abcam, UK) using Pierce™ Protein A/G Magnetic Beads (Thermo Scientific) and the released proteins (rTcpC, PAD4 and ubiquitin) were detected by immunoblotting. In detection of ubiquitin and rTcpC, rabbit anti-ubiquitin IgG (1:1000 dilution, Abcam, UK) and the self-made rabbit anti-rTcpC IgG were used as the primary antibody respectively, and IRDye® 680RD goat anti-rabbit-IgG (H + L) (1:5000 dilution, LI-COR, USA) as secondary antibody.

### In vitro ubiquitination kit assays

MuRF1 Ubiquitin Ligase Kit-S5a Substrate (Boston Biochem, USA) was used to detect ubiquitination of PAD4 and determine the E3 ligase function of rTcpC. When the E3 was replaced by rTcpC, proteins in the neutrophil lysates and rPAD4 (Abcam, UK) were used as the substrates. All the experiments were performed according to instructions of the manufacturer. Ubiquitination of PAD4 was detected by western blotting as described above.

### Confocal microscopy to examine co-localization of PAD4 with PSMD2

Neutrophils were treated with or without 4 μg/ml rTcpC and 1 μg/ml LPS for the indicated time, cells were collected and fixed with 4% paraformaldehyde overnight at 4 °C. The fixed cells were permeabilized with 0.1% TritonX-100 in PBS for 20 min, and washed three times with PBS. After blocked with 1% BSA, 0.1% Tween 20 in PBS for 2 h at room temperature, cells were stained with rabbit anti-PAD4 IgG (1:100 dilution, Abcam, UK) overnight at 4 °C, and then probed with the secondary antibody Alex Flour 647 goat anti-rabbit IgG fluorescence antibody (1:500 dilution, Invitrogen, USA) for 2 h at room temperature in dark. After three times wash with PBS, the cells were also probed with rabbit anti-PSMD2 (1:100 dilution, Abcam, UK) and Alexa Fluor 488 goat anti-rabbit IgG fluorescent antibody (1:200 dilution, Invitrogen, USA) as primary and secondary antibodies respectively. Neutrophils were finally washed with PBS and stained with 100 ng/ml DAPI for 30 min. Co-localization of PAD4 with PSMD2 was observed by confocal microscopy (Olympus) (495/519, 652/668 or 485 nm excitation/emission wavelengths for Alexa Fluor 488, Alexa Fluor 647 or DAPI detection). Percentages and yellow FI reflecting the co-localization were analyzed by ImageJ software^62^. To examine the influence of MG-132 on rTcpC induced accumulation of PAD4 in proteasomes, neutrophils were treated with 1 μM MG-132 for 30 min before treatment with 4 μg/ml rTcpC for the indicated time, and then the treated cells were subjected to confocal microscopy as described above.

### Transcriptome sequencing

Neutrophils (1 × 10^6^ cells) were treated for 120 min with 1 μg/ml LPS, 4 μg/ml rTcpC, 4 μg/ml rTcpC + 1 μg/ml LPS, respectively, and untreated neutrophils were served as the control group. Cells were collected (1500 rpm, 10 min) and frozen at −80 °C with RNAiso Plus (TaKaRa, Japan). Total RNA was extracted and a genome-wide transcriptomics analysis was conducted (LC-BIOTECHNOLOGIS (HANGZHOU) CO., LTD). The differentially expressed mRNAs were selected with fold change >2 or fold change <0.5 and *p*-value < 0.05 by R package edgeR (https://bioconductor.org/packages/release/bioc/html/edgeR.html) or DESeq2 (http://www.bioconductor.org/packages/release/bioc/html/DESeq2.html).

### Bioinformatics analyses

The structure and functional domains in PAD4 of human and mouse were analyzed using NCBI-Batch CD-Search software^63^.

### Statistics and reproducibility

Data shown are mean ± SD of three independent experiments. Images were analyzed by ImageJ software. Dunnett and Mann–Whitney tests of SPSS 22.0 were used for variance analysis. Data from quantification of NETs formation were analyzed by Mann–Whitney test. *p* < 0.05 is considered to be statistically significant and *p* < 0.01 is extremely significant. NS is considered to be not significant. All source data are provided in separate Raw Data file of Source Data file.

### Reporting summary

Further information on experimental design is available in the Nature Research Reporting Summary linked to this paper.

## Supplementary information

Supplementary Information

Reporting summary

## Data Availability

Data supporting the findings of this manuscript are available from the corresponding author upon request. Full scan images of the Gels and Blots and Source data for figures and numbers are provided with this paper. We deposited our data including the transcriptomic data in GEO DataSets, the accession number is GSE173807. Source data are provided with this paper.

## Source data

Source data
