# Supplementary material for: TcpC inhibits neutrophil extracellular trap formation by enhancing ubiquitination mediated degradation of peptidylarginine deiminase 4
Source: Nat Commun. 2021 Jun 9;12:3481. doi: 10.1038/s41467-021-23881-8 (PMC8190435; doi:10.1038/s41467-021-23881-8)
Supplement: Supplementary file 1 — Supplementary Information [file 41467_2021_23881_MOESM1_ESM.pdf]

# TcpC inhibits neutrophil extracellular trap formation by enhancing ubiquitination mediated degradation of peptidylarginine deiminase 4

# Supplementary information

## Supplementary Methods

**Verification of rTcpC entry into neutrophils.** $1\times10^6$/ml neutrophils were incubated with 4 μg/ml rTcpC and were collected at indicated time during the period of 120 min. Cells were subjected to confocal observation and Western blot analyses of rTcpC, respectively. For confocal analyses of rTcpC entry into neutrophils, the treated neutrophils were fixed by 4% paraformaldehyde in PBS overnight at 4°C, permeabilized with 0.1% TritonX-100 in PBS for 20 min, and washed three times with PBS. After blocked with 1% BSA, 0.1% Tween 20 in PBS for 2 h at room temperature, the cells were probed with rabbit anti-Ly-6G-IgG (Abcam, UK) overnight at 4°C. Then, the cells were washed three times with PBS and incubated in the dark with goat anti-rabbit IgG antibody (Alex Flour 647 nm, 1:500 diluted in the PBS) for 2 h at room temperature. After three times wash with PBS, the cells were incubated with rabbit anti-rTcpC (made in our laboratory) overnight at 4°C. After three times wash with PBS, the cells were stained with Alex Flour 488 nm goat anti-rabbit IgG fluorescent secondary antibody for 2 h at room temperature. Finally, DAPI and ProLong Diamond Antifade Mountant (Thermo, USA) were added to each coverslip, and the cells were observed under a confocal microscope (FV3000

Olympus, Japan). Dynamic analyses of rTcpC in the treated neutrophils by Western blot were performed as described in the text.

**Examination of endocytic vesicle mediating the entry of rTcpC into neutrophils.** To determine the endocytic vesicle that mediates the entry of rTcpC into neutrophils, $1 \times 10^6$/ml neutrophils were pre-treated with 50 μM MCD[1,2] or 80 μM dynasore[3] or the solvent DMSO for 30 min, then incubated with 4 μg/ml rTcpC for 120 min. The cells were collected and used for confocal and Western blot analyses of rTcpC as described above.

**Identification of neutrophils by Flow cytometry.** Human neutrophils were isolated by EasySep Direct Human Neutrophil Isolation Kit and the purification was analyzed by FACS using Ly-6G/Ly-6C monoclonal antibody (1:500 dilution, PE, Thermo, USA) and CD11b monoclonal antibody (1:500 dilution, FITC, Thermo, USA).

# Supplementary Figures

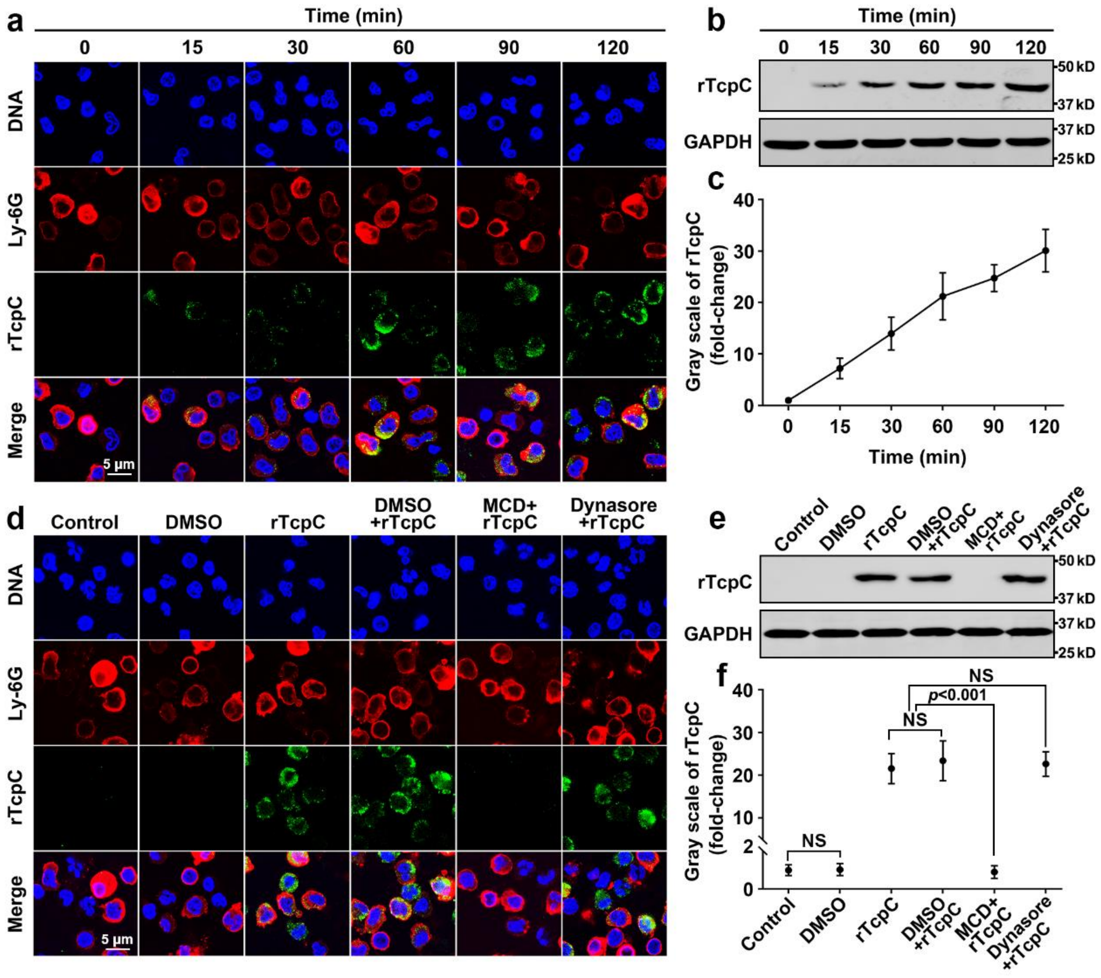


**Supplementary Figure 1. rTcpC can get into neutrophils through caveolin-mediated endocytosis. a** Dynamic observation of rTcpC entry into neutrophils by Confocal, Scale bar =5 μm. **b** Dynamic analyses of rTcpC protein levels in rTcpC-treated neutrophils by Western blot. Confocal images and western blots are representative of 3 independent experiments, n=3. **c** Gray scale analyses of rTcpC bands in experiments described in **b**. **d** Confocal observation of the influence of endocytosis inhibitor MCD or dynasore on entry of rTcpC into neutrophils. Confocal images are representative of 3 independent experiments, n=3. Scale bar =5 μm. **e** Western blot analyses of rTcpC in treated neutrophils. **f** Gray scale analyses of

rTcpC bands from experiments as described in **e**. Mean ± SD of three independent experiments were shown. $p<0.01$ was considered to be statistically significant. NS: not significant. *p*-values were derived by Dunnett and Mann-Whitney tests. All Western blots in panels **b** and **c** are provided as uncropped blots in Gels and Blots of Source Data file. Source data for panel **c** and **f** are provided in the separate Source Data file.

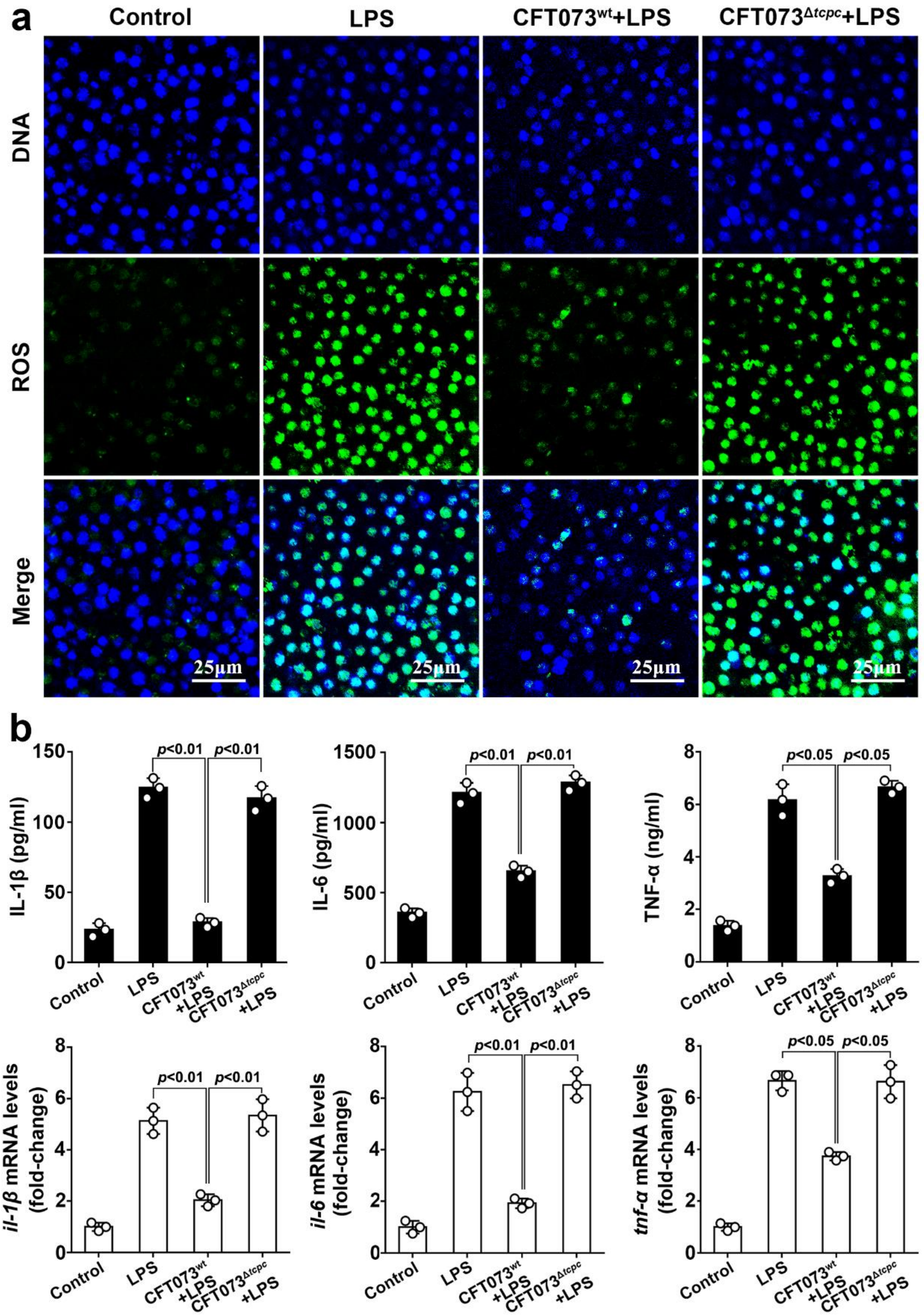


**Supplementary Figure 2. CFT073$^{wt}$ inhibits production of ROS and inflammatory cytokines in LPS-induced NETosis. a** Confocal analyses of the influence of CFT073$^{wt}$ on LPS-induced ROS. Images are representative of 3

experiments, n=3. Scale bar=25 μm. **b** Protein and mRNA levels of IL-1β, IL-6, TNF-α were detected by ELISA and qRT-PCR. Mean ± SD of three independent experiments were shown. $p<0.05$ and $p<0.01$ were considered to be statistically significant and extremely significant respectively. *p*-values were derived by Dunnett and Mann-Whitney multiple comparison tests. Source data for panel **b** are provided in the separate Source Data file.

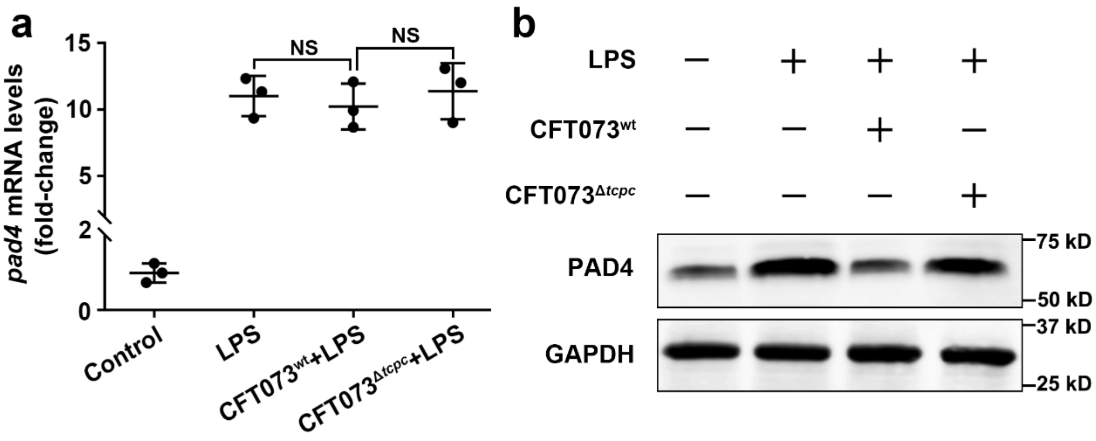


**Supplementary Figure 3. CFT073$^{wt}$ decreases protein but not mRNA levels of PAD4. a** qRT-PCR to examine mRNA levels of PAD4 in different groups of neutrophils. Mean ± SD of three independent experiments were shown. NS: not significant. **b** Western blot analyses of PAD4 protein levels in neutrophils treated with CFT073$^{wt}$ or CFT073$^{\Delta tcpc}$. Western blots are representative of 3 biological repeats, n=3. All Western blots are provided as uncropped blots in Gels and Blots of Source Data file. Source data for panel **a** are provided in the separate Source Data file.

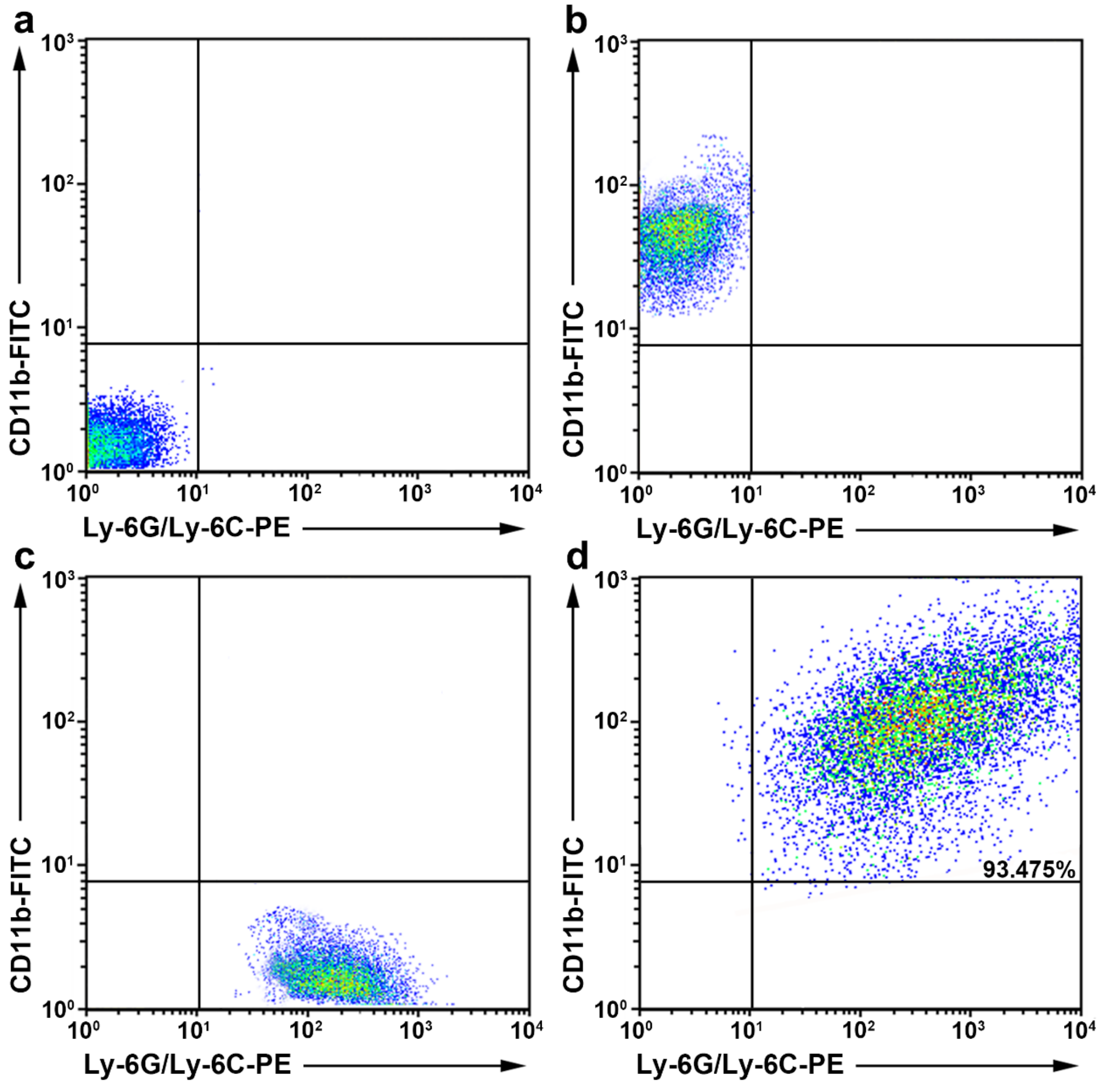


**Supplementary Figure 4. Purity of human neutrophils.** Purity of the human neutrophils was evaluated by FACS using antibodies against Ly-6G/Ly-6C and CD11b. **a** Isotype control. **b-c** Cells were singly stained with PE-Ly-6G/Ly-6C monoclonal antibody (FL-2 channel) and FITC-CD11b monoclonal antibody (FL-1 channel), respectively. **d** Cells were double stained with FITC-anti CD11b and PE-anti Ly6G.

## Supplementary References

1. Cullen, P. J. & Steinberg, F. To degrade or not to degrade: mechanisms and

significance of endocytic recycling. *Nat. Rev. Mol. Cell Biol.* **19**, 679-696, doi:10.1038/s41580-018-0053-7 (2018).

2. McMahon, H. T. & Boucrot, E. Molecular mechanism and physiological functions of clathrin-mediated endocytosis. *Nat. Rev. Mol. Cell Biol.* **12**, 517-533, doi:10.1038/nrm3151 (2011).

3. Almeida-Souza, L. et al. A Flat BAR Protein Promotes Actin Polymerization at the Base of Clathrin-Coated Pits. *Cell* **174**, 325-337 .e14, doi:10.1016/j.cell.2018.05.020 (2018).
