# Supplementary material for: TcpC inhibits neutrophil extracellular trap formation by enhancing ubiquitination mediated degradation of peptidylarginine deiminase 4
Source: Nat Commun. 2021 Jun 9;12:3481. doi: 10.1038/s41467-021-23881-8 (PMC8190435; doi:10.1038/s41467-021-23881-8)
Supplement: Supplementary file 2 — Reporting summary [file 41467_2021_23881_MOESM2_ESM.pdf]

nature research

Corresponding author(s): NCOMMS-20-30811

Last updated by author(s): May 5, 2021

Reporting Summary

Nature Research wishes to improve the reproducibility of the work that we publish. This form provides structure for consistency and transparency in reporting. For further information on Nature Research policies, see our Editorial Policies and the Editorial Policy Checklist.

Statistics

For all statistical analyses, confirm that the following items are present in the figure legend, table legend, main text, or Methods section.

n/a	Confirmed	
☐	☒	The exact sample size (*n*) for each experimental group/condition, given as a discrete number and unit of measurement
☐	☒	A statement on whether measurements were taken from distinct samples or whether the same sample was measured repeatedly
☐	☒	The statistical test(s) used AND whether they are one- or two-sided<br>*Only common tests should be described solely by name; describe more complex techniques in the Methods section.*
☐	☒	A description of all covariates tested
☐	☒	A description of any assumptions or corrections, such as tests of normality and adjustment for multiple comparisons
☐	☒	A full description of the statistical parameters including central tendency (e.g. means) or other basic estimates (e.g. regression coefficient) AND variation (e.g. standard deviation) or associated estimates of uncertainty (e.g. confidence intervals)
☐	☒	For null hypothesis testing, the test statistic (e.g. *F*, *t*, *r*) with confidence intervals, effect sizes, degrees of freedom and *P* value noted<br>*Give P values as exact values whenever suitable.*
☒	☐	For Bayesian analysis, information on the choice of priors and Markov chain Monte Carlo settings
☒	☐	For hierarchical and complex designs, identification of the appropriate level for tests and full reporting of outcomes
☒	☐	Estimates of effect sizes (e.g. Cohen's *d*, Pearson's *r*), indicating how they were calculated

Our web collection on statistics for biologists contains articles on many of the points above.

Software and code

Policy information about availability of computer code

Data collection	SPSS 22.0 were used for variance analysis, Images were analyzed by ImageJ software, GraphPad Prism 7.00
Data analysis	Dunnett and Mann-Whitney tests of SPSS 22.0 were used for variance analysis. Data from quantification of NETs formation were analyzed by Mann-Whitney test. p<0.05 is considered to be statistically significant and p<0.01 is extremely significant. NS is considered to be not significant. All source data are provided in separate Raw Data file of Source Data file.

For manuscripts utilizing custom algorithms or software that are central to the research but not yet described in published literature, software must be made available to editors and reviewers. We strongly encourage code deposition in a community repository (e.g. GitHub). See the Nature Research guidelines for submitting code & software for further information.

Data

Policy information about availability of data

All manuscripts must include a data availability statement. This statement should provide the following information, where applicable:

- Accession codes, unique identifiers, or web links for publicly available datasets
- A list of figures that have associated raw data
- A description of any restrictions on data availability

Data supporting the findings of this manuscript are available from the corresponding author upon request. Full scan images of the Gels and Blots and Source data for figures and numbers are provided with this paper.

Field-specific reporting

Please select the one below that is the best fit for your research. If you are not sure, read the appropriate sections before making your selection.

☒ Life sciences ☐ Behavioural & social sciences ☐ Ecological, evolutionary & environmental sciences

For a reference copy of the document with all sections, see nature.com/documents/nr-reporting-summary-flat.pdf

Life sciences study design

All studies must disclose on these points even when the disclosure is negative.

Sample size	Gross pathological observation of kidneys from CFT073wt- or CFT073tcpc- induced pyelonephritis mouse models. One representative kidney from five mice in each group was shown. n=5<br>Histological examination of kidneys from CFT073wt- or CFT073Δtcpc-induced pyelonephritis mouse models. Scale bar=25 μm. One representative image of kidneys from 5 mice in each group was shown, n=3.<br>Mean ± SD of three independent experiments were shown, n=3
Data exclusions	Exclude abnormal data that are too large or too small compared with the average value in the experiment
Replication	n≥3
Randomization	Experiment was repeated at least 3 times, and the results of 3 times were randomly selected for analysis
Blinding	In the process of data collection and analysis, researchers have a clear understanding of the experimental grouping, which can make us better analyze and count our data

Reporting for specific materials, systems and methods

We require information from authors about some types of materials, experimental systems and methods used in many studies. Here, indicate whether each material, system or method listed is relevant to your study. If you are not sure if a list item applies to your research, read the appropriate section before selecting a response.

Materials & experimental systems

n/a	Involved in the study	
☐	☒	Antibodies
☒	☐	Eukaryotic cell lines
☒	☐	Palaeontology and archaeology
☐	☒	Animals and other organisms
☐	☒	Human research participants
☒	☐	Clinical data
☒	☐	Dual use research of concern

Methods

n/a	Involved in the study	
☐	☒	ChIP-seq
☐	☒	Flow cytometry
☒	☐	MRI-based neuroimaging

Antibodies

Antibodies used	rabbit anti-PAD4 IgG (1:100 dilution, Abcam, UK), Alex Flour 647 goat anti-rabbit IgG fluorescence antibody (1:500 dilution, Invitrogen, USA), rabbit anti-PSMD2 (1:100 dilution, Abcam, UK), Alexa Fluor 488 goat anti-rabbit IgG fluorescent antibody (1:200 dilution, Invitrogen, USA) , rabbit anti-ubiquitin IgG (1:1000 dilution, Abcam, UK), self-made rabbit anti-rTcpC IgG, IRDye® 680RD goat anti-rabbit-IgG (H+L) (1:5000 dilution, LI-COR, USA), immunoprecipitated with the rabbit anti-PAD4 IgG (1:1000 dilution, Abcam, UK) using Pierce™ Protein A/G Magnetic Beads (Thermo Scientific), rabbit anti-CitH3 IgG (1:5000 dilution, Abcam, UK) as primary antibody and IRDye® 680RD goat anti-rabbit-IgG (H+L) (1:5000 dilution, LI-COR, USA), rabbit anti-PAD4 IgG (1:10000 dilution, Abcam, UK) as primary antibody and IRDye® 680RD goat anti-rabbit-IgG (H+L) (1:5000 dilution, LI-COR, USA) as secondary antibody. rabbit anti-MPO antibodies (Abcam, UK, 1:200 diluted in 0.1% Tween 20 in PBS). rabbit anti-CitH3 antibodies (1:200 dilution, Abcam, UK, 1:200 diluted in 0.1% Tween 20 in PBS). Ly-6G/Ly-6C (1:500 dilution, Ly-6G/Ly-6C monoclonal antibody, PE, Thermo, USA) and CD11b (1:500 dilution, CD11b monoclonal antibody, FITC, Thermo, USA).
Validation	The fixed tissues were incubated with rabbit anti-Ly-6G-IgG, rabbit anti-CitH3-IgG, and rabbit anti-MPO-IgG (Abcam, UK, 1:200 dilution) overnight at 4C and stained with different fluorescent secondary antibody (Alexa fluor 546 nm goat anti-rabbit IgG, Alexa fluor 488nm goat anti-rabbit IgG and Alexa fluor 647nm goat anti-rabbit IgG, 1:500 dilution) in dark respectively.<br>Purity of the human neutrophils was evaluated by FACS using antibodies against Ly-6G/Ly-6C (1:500 dilution, Ly-6G/Ly-6C monoclonal antibody, PE, Thermo, USA) and CD11b (1:500 dilution, CD11b monoclonal antibody, FITC, Thermo, USA).<br>Confocal microscopy to examine NETosis:rabbit anti-MPO antibodies (Abcam, UK, 1:200 diluted in 0.1% Tween 20 in PBS) overnight at 4C. Next, neutrophils were gently washed three times with PBS and incubated in the dark with goat anti-rabbit IgG antibody (Alex Flour 647 nm, 1:500 diluted in the PBS) for 2 h at room temperature.neutrophils were incubated with rabbit anti-CitH3 antibodies (1:200 dilution, Abcam, UK, 1:200 diluted in 0.1% Tween 20 in PBS) overnight at 4C, then washed three times and incubated with

Alexa fluor 488 nm goat anti-rabbit IgG fluorescent secondary antibody.
Western Blot analyses of PAD4.Proteins were probed with rabbit anti-PAD4 IgG (1:10000 dilution, Abcam, UK) as primary antibody and IRDye® 680RD goat anti-rabbit-IgG (H+L) (1:5000 dilution, LI-COR, USA) as secondary antibody.
Dynamic analyses of CitH3 levels.Proteins were probed with rabbit anti-CitH3 IgG (1:5000 dilution, Abcam, UK) as primary antibody and IRDye® 680RD goat anti-rabbit-IgG (H+L) (1:5000 dilution, LI-COR, USA).
Co-immunoprecipitation and immunoblotting to detect ubiquitination of PAD4. rabbit anti-PAD4 IgG (1:1000 dilution, Abcam, UK) using Pierce™ Protein A/G Magnetic Beads (Thermo Scientific) and the released proteins (rTcpC, PAD4 and ubiquitin) were detected by immunoblotting. In detection of ubiquitin and rTcpC, rabbit anti-ubiquitin IgG (1:1000 dilution, Abcam, UK) and the self-made rabbit anti-rTcpC IgG were used as the primary antibody respectively, and IRDye® 680RD goat anti-rabbit-IgG (H+L) (1:5000 dilution, LI-COR, USA) as secondary antibody.
Confocal microscopy to examine co-localization of PAD4 with PSMD2. cells were stained with rabbit anti-PAD4 IgG (1:100 dilution, Abcam, UK) overnight at 4C,   and then probed with the secondary antibody Alex Flour 647 goat anti-rabbit IgG fluorescence antibody (1:500 dilution, Invitrogen, USA) for 2 h at room temperature in dark. After three times wash with PBS, the cells were also probed with rabbit anti-PSMD2 (1:100 dilution, Abcam, UK) and Alexa Fluor 488 goat anti-rabbit IgG fluorescent antibody (1:200 dilution, Invitrogen, USA) as primary and secondary antibodies respectively.

Animals and other organisms

Policy information about studies involving animals; ARRIVE guidelines recommended for reporting animal research

Laboratory animals	Female C57BL/6 mice, 6~8 weeks of age, were provided by Shanghai Slack Laboratory Animals Co., Ltd (Shanghai, China).
Wild animals	Not used
Field-collected samples	72 hours later, the model mice were sacrificed and kidneys were removed for pathological and in situ NETosis examinations as described previously
Ethics oversight	Animal experiments were performed in accordance with the National Regulations for the Administration of Experimental Animals of China (1988–002) and the National Guidelines for Experimental Animal Welfare of China (2006–398).

Note that full information on the approval of the study protocol must also be provided in the manuscript.

Human research participants

Policy information about studies involving human research participants

Population characteristics	20 donors
Recruitment	Voluntary blood donation
Ethics oversight	All the animal experimental protocols were approved by the Ethics Committee for Animal Experiment of Zhejiang University City College School of Medicine and fully informed consent of all healthy blood donors was obtained after clearing the possible consequences of the study.

Note that full information on the approval of the study protocol must also be provided in the manuscript.

ChIP-seq

Data deposition

- [x] Confirm that both raw and final processed data have been deposited in a public database such as GEO.
- [ ] Confirm that you have deposited or provided access to graph files (e.g. BED files) for the called peaks.

Data access links
May remain private before publication.

https://www.ncbi.nlm.nih.gov/geo/query/acc.cgi?acc=GSE173807

Files in database submission

1_gene_expression_statistics
1_transcript_expression_statistics
2_transcript_expression_interval
2_gene_expression_interval
3_transcript_expression_coverage
4_genes_fpkm_expression
5_isoforms_fpkm_expression
gene_count_matrix
differentially_expressed_genes
ReadsQC
0_gene_annotation
1_summary_of_gene
2_mapped_stat
3_mapped_region_stat

merged.fa
1_SNV_INDEL_Statistics
Control1_Clean_Data2.fq fastq
LPS1_Clean_Data1.fq fastq
LT1_Clean_Data1.fq fastq
T1_Clean_Data1.fq fastq
2_SNV_INEDL_Position_Type
3_SNP_Variation_Type
4_SNV_INEDL_Annotation
5_SNV_All_Samples
6_INDEL_All_Samples
transcript_count_matrix

Genome browser session (e.g. UCSC)

GEO Datasets

Methodology

Replicates

Data are representative of 3 biological repeats (n=3).

Sequencing depth

Coverage Control1 Control2 Control3 LPS1 LPS2 LPS3 LT1 LT2 LT3 T1 T2 T3
0-1 35.13% 33.10% 33.25% 34.49% 33.92% 35.73% 28.63% 29.72% 31.31% 28.49% 29.40% 30.43%
2-5 33.98% 33.11% 33.68% 33.62% 33.48% 33.82% 32.33% 32.58% 33.51% 32.87% 32.68% 33.02%
6-10 11.53% 11.46% 11.87% 11.45% 11.68% 11.17% 13.03% 12.97% 12.84% 13.13% 13.18% 12.74%
11-15 5.10% 5.34% 5.34% 5.33% 5.26% 5.10% 6.51% 6.50% 6.11% 6.68% 6.45% 6.25%
16-20 3.01% 3.34% 3.19% 3.11% 3.14% 2.94% 4.13% 3.85% 3.68% 4.14% 4.00% 3.72%
21-25 2.00% 2.16% 2.19% 2.01% 2.13% 1.97% 2.70% 2.61% 2.34% 2.76% 2.70% 2.53%
26-30 1.43% 1.52% 1.58% 1.51% 1.50% 1.42% 2.01% 1.86% 1.66% 1.97% 1.82% 1.81%
>30 7.82% 9.97% 8.91% 8.48% 8.91% 7.86% 10.65% 9.92% 8.55% 9.96% 9.77% 9.49%

Antibodies

Dynabeads Oligo (dT), 25-61005, Thermo Fisher, USA
NEBNext® Magnesium RNA Fragmentation Module, E6150S, USA
Invitrogen SuperScript™ II Reverse Transcriptase, 1896649, CA, USA
E. coli DNA polymerase I NEB, m0209, USA
RNase H NEB, m0297, USA
dUTP Solution Thermo Fisher, R0133, CA, USA
UDG NEB, m0280, MA, US

Peak calling parameters

Sample Valid reads Mapped reads Unique Mapped reads Multi Mapped reads PE Mapped reads Reads map to sense strand Reads map to antisense strand Non-splice reads Splice reads
Control1 31785418 30594851(96.25%) 25554525(80.40%) 5040326(15.86%) 24860468(78.21%) 14881666(46.82%) 14915711 (46.93%) 19044287(59.92%) 10753090(33.83%)
Control2 44785014 43365839(96.83%) 35966890(80.31%) 7398949(16.52%) 35123124(78.43%) 21066846(47.04%) 21108024 (47.13%) 26570143(59.33%) 15604727(34.84%)
Control3 38017612 36749789(96.67%) 30910223(81.31%) 5839566(15.36%) 27184916(71.51%) 17912904(47.12%) 17950242 (47.22%) 23396156(61.54%) 12466990(32.79%)
LPS1 35694566 34558308(96.82%) 29089549(81.50%) 5468759(15.32%) 25321914(70.94%) 16827643(47.14%) 16861128(47.24%) 22051110(61.78%) 11637661(32.60%)
LPS2 38303156 36949256(96.47%) 30778216(80.35%) 6171040(16.11%) 28686188(74.89%) 17974464(46.93%) 18010736(47.02%) 23098101(60.30%) 12887099(33.65%)
LPS3 31420640 30350161(96.59%) 25400601(80.84%) 4949560(15.75%) 22098602(70.33%) 14771085(47.01%) 14798539(47.10%) 19064180(60.67%) 10505444(33.43%)
LT1 46432500 44913229(96.73%) 35302596(76.03%) 9610633(20.70%) 34602530(74.52%) 21243322(45.75%) 21291640(45.86%) 28167166(60.66%) 14367796(30.94%)
LT2 41392716 39914027(96.43%) 31463347(76.01%) 8450680(20.42%) 30843860(74.52%) 18961359(45.81%) 19008538(45.92%) 24804723(59.93%) 13165174(31.81%)
LT3 34366110 33180264(96.55%) 25838711(75.19%) 7341553(21.36%) 23969404(69.75%) 15716267(45.73%) 15750945(45.83%) 20128623(58.57%) 11338589(32.99%)
T1 41426698 40063803(96.71%) 31726705(76.59%) 8337098(20.12%) 28414288(68.59%) 19002615(45.87%) 19043477(45.97%) 25570106(61.72%) 12475986(30.12%)
T2 41466642 39969634(96.39%) 31493964(75.95%) 8475670(20.44%) 30494472(73.54%) 18889647(45.55%) 18938836(45.67%) 25164063(60.69%) 12664420(30.54%)
T3 40830886 39404633(96.51%) 30681526(75.14%) 8723107(21.36%) 32854758(80.47%) 18702448(45.80%) 18753705(45.93%) 24441197(59.86%) 13014956(31.88%)

Data quality

After the final transcriptome was generated, StringTie and ballgown(http://www.bioconductor.org/packages/release/bioc/html/ballgown.html) were used to estimate the expression levels of all transcripts and perform expression level for mRNAs by calculating FPKM (FPKM = [total_exon_fragments / mapped_reads(millions) × exon_length(kB)]),(command line: ~stringtie -e -B -p 4 -G

merged.gtf -o samples.gtf samples.bam). The differentially expressed mRNAs were selected with fold change > 2 or fold change < 0.5 and p value < 0.05 by R package edgeR(https://bioconductor.org/packages/release/bioc/html/edgeR.html) or DESeq2(http://www.bioconductor.org/packages/release/bioc/html/DESeq2.html),and then analysis GO enrichment and KEGG enrichment to the differentially expressed mRNAs.

Software

https://cutadapt.readthedocs.io/en/stable/cutadapt-1.9
https://daehwankimlab.github.io/hisat2/
http://ccb.jhu.edu/software/stringtie/
http://ccb.jhu.edu/software/stringtie/gffcompare.shtml
https://bioconductor.org/packages/release/bioc/html/edgeR.html
http://www.bioconductor.org/packages/release/bioc/html/DESeq2.html

Flow Cytometry

Plots

Confirm that:

- [x] The axis labels state the marker and fluorochrome used (e.g. CD4-FITC).
- [x] The axis scales are clearly visible. Include numbers along axes only for bottom left plot of group (a 'group' is an analysis of identical markers).
- [ ] All plots are contour plots with outliers or pseudocolor plots.
- [x] A numerical value for number of cells or percentage (with statistics) is provided.

Methodology

Sample preparation

Purity of the human neutrophils was evaluated by FACS using antibodies against Ly-6G/Ly-6C (1:500 dilution, Ly-6G/Ly-6C monoclonal antibody, PE, Thermo, USA) and CD11b (1:500 dilution, CD11b monoclonal antibody, FITC, Thermo, USA). Neutrophil purity was >93% (Supplementary Fig. 4).

Instrument

BD FACSCalibur

Software

FlowJo_V10

Cell population abundance

Neutrophil purity was >93% (Supplementary Fig. 4).

Gating strategy

We provide the information of purity of the human neutrophils was evaluated by FACS using antibodies against Ly-6G/Ly-6C and CD11b in supplementary information method. And We also provided the supplementary figure of Isotype control, cells were singly stained with PE-Ly-6G/Ly-6C monoclonal antibody (FL-2 channel) and FITC-CD11b monoclonal antibody (FL-1 channel), respectively, and cells were double stained with FITC-anti CD11b and PE-anti Ly6G.

- [x] Tick this box to confirm that a figure exemplifying the gating strategy is provided in the Supplementary Information.
